# Clinical and Lifestyle Factors and Risk of Amyotrophic Lateral Sclerosis: A Population-Based Case-Control Study

**DOI:** 10.3390/ijerph17030857

**Published:** 2020-01-30

**Authors:** Tommaso Filippini, Maria Fiore, Marina Tesauro, Carlotta Malagoli, Michela Consonni, Federica Violi, Elisa Arcolin, Laura Iacuzio, Gea Oliveri Conti, Antonio Cristaldi, Pietro Zuccarello, Elisabetta Zucchi, Letizia Mazzini, Fabrizio Pisano, Ileana Gagliardi, Francesco Patti, Jessica Mandrioli, Margherita Ferrante, Marco Vinceti

**Affiliations:** 1 CREAGEN—Environmental, Genetic and Nutritional Epidemiology Research Center, Department of Biomedical, Metabolic and Neural Sciences, University of Modena and Reggio Emilia, 41125 Modena, Italy; tommaso.filippini@unimore.it (T.F.); carlotta.malagoli@unimore.it (C.M.); federica.violi@unimore.it (F.V.); elisa.arcolin@gmail.com (E.A.); l.iacuzio@ausl.mo.it (L.I.); 2 Clinical and Experimental Medicine PhD Program, University of Modena and Reggio Emilia, 41125 Modena, Italy; 3 Department of Medical, Surgical Sciences and Advanced Technologies “G. F. Ingrassia”, Catania University, 95123 Catania, Italy; mfiore@unict.it (M.F.); olivericonti@unict.it (G.O.C.); antonio.cristaldi81@gmail.com (A.C.); pietro.zuccarello@unict.it (P.Z.); patti@unict.it (F.P.); marfer@unict.it (M.F.); 4 Department of Biomedical, Surgical and Dental Sciences, University of Milan, 20122 Milan, Italy; marina.tesauro@unimi.it (M.T.); michela.consonni@unimi.it (M.C.); 5 Azienda USL-IRCCS di Reggio Emilia, 42122 Reggio Emilia, Italy; 6 Department of Public Health, Local Health Unit, 41121 Modena, Italy; 7 Neurology Unit, Department of Biomedical, Metabolic and Neural Sciences, University of Modena and Reggio Emilia, 41125 Modena, Italy; elibettizucchi@gmail.com; 8 ALS Centre Department of Neurology, ‘Maggiore della Carità’ University Hospital, 28100 Novara, Italy; letizia.mazzini@uniupo.it (L.M.); ileanagagliardi91@gmail.com (I.G.); 9 Neurological Rehabilitation Division, Policlinico San Marco di Zingonia, 24046 Zingonia (BG), Italy; fabrizio.pisano@grupposandonato.it; 10 Neurology Unit, Department of Neuroscience, S. Agostino Estense Hospital, Azienda Ospedaliero Universitaria di Modena, 41126 Modena, Italy; mandrioli.jessica@aou.mo.it; 11 Department of Epidemiology, Boston University School of Public Health, Boston, MA 02118, USA

**Keywords:** amyotrophic lateral sclerosis, case-control study, environmental factors, trauma, diet

## Abstract

*Background*: Amyotrophic lateral sclerosis (ALS) is a progressive, fatal neurodegenerative disease of the motor neurons. The etiology of ALS remains largely unknown, particularly with reference to the potential environmental determinants. *Methods*: We performed a population-based case-control study in four provinces from both Northern and Southern Italy in order to assess non-genetic ALS risk factors by collecting through tailored questionnaires information about clinical and lifestyle factors. We estimated ALS risk by calculating odds ratio (OR) with its 95% confidence interval (CI) using unconditional logistic regression models adjusted for sex, age and educational attainment. *Results*: We recruited 230 participants (95 cases and 135 controls). We found a possible positive association of ALS risk with trauma, particularly head trauma (OR = 2.61, 95% CI 1.19–5.72), electric shock (OR = 2.09, 95% CI 0.62–7.06), and some sports, although at a competitive level only. In addition, our results suggest an increased risk for subjects reporting use of private wells for drinking water (OR = 1.38, 95% CI 0.73–2.27) and for use of herbicides during gardening (OR = 1.95, 95% CI 0.88–2.27). Conversely, there was a suggestion of an inverse association with overall fish consumption (OR = 0.27, 95% CI 0.12–0.60), but with no dose-response relation. Consumption of some dietary supplements, namely those containing amino acids and, in the Southern Italy population, vitamins and minerals such as selenium, seemed associated with a statistically imprecise increased risk. *Conclusions*: Our results suggest a potential etiologic role a number of clinical and lifestyle factors with ALS risk. However, caution is needed due to some study limitations. These include the small sample size and the low number of exposed subjects, which affect statistical precision of risk estimates, the potential for exposure misclassification, and the uncertainties about mechanisms underpinning the possible association between these factors and disease risk.

## 1. Introduction

Amyotrophic lateral sclerosis (ALS) is a neurodegenerative disease that affects both upper and lower motor neurons. Disease progression is generally rapid and, after substantial respiratory and nutritional failure, leads to death in 70–80% of affected individuals within 5 years [1,2,3]. The worldwide prevalence of ALS is approximately 6 cases per 100,000 individuals [4], while annual incidence varies worldwide from approximately 1 to 2.6 cases per 100,000 individuals. In terms of geographical distribution, incidence is higher in populations of European than Asian origin [5]. An increase in ALS incidence has been reported in recent decades [6], in Italy as well [7,8,9,10,11]. Despite progress in assessing the role of genetic factors [12], the etiology of ALS remains largely unknown. To date, the only recognized risk factors are male sex, older age and a family history of ALS, which is frequently associated with specific gene mutations [13]. Many putative exogenous factors have been investigated, including exposure to pesticides, electromagnetic fields, some metals, cyanobacteria contamination, as well as a history of medical conditions and lifestyle choices [14,15,16,17,18,19]. Nevertheless, a definite environmental risk factor has not been identified so far. In the present study, we aimed at investigating the association between clinical history, lifestyles, and other personal characteristics with the risk of ALS using a large population-based case-control study in four Italian provinces.

## 2. Methods

Subsequent to approval by the Ethics Committees of Catania, Modena and Reggio Emilia (no. 80/11), and Novara (no. 4/12), we designed a population-based case-control study to investigate the role of clinical and environmental factors in the etiology of ALS. We attempted to recruit all sporadic ALS cases diagnosed in the period 2008–2011 in the provinces of Catania, Modena, and Reggio Emilia, and in the period 2002–2012 in Novara province.

We used multiple sources for the identification of eligible cases: the Emilia-Romagna Registry for ALS (ERRALS, established in 2009), and the Piedmont and Valle d’Aosta Register for ALS (PARALS, established in 1995), including hospital discharge records for the entire study period, death certificate files, and the drug prescription directories of Emilia-Romagna, Piedmont and Sicily. We only included in the study patients diagnosed with ‘definite’ and ‘probable’ ALS based on the El Escorial revised criteria. We therefore excluded familial cases [20]. Finally, we tested cases for repeat expansions in the *C9orf72* gene.

Using the National Health Service directory of the residents in the study provinces, we randomly selected controls from four populations, matched by sex, age (+/−5) and province of residence. We recruited cases either at the Neurology Units of the study area or by contacting them by phone and/or regular-mail. Study materials included an information leaflet with the description of study aim, methods and contact details of the study investigators, the informed consent form, the questionnaire, and a prepaid return envelope for those contacted by mail.

We estimated ALS risk by calculating the disease odds ratio (OR) and 95 % confidence intervals (CI) in crude and adjusted unconditional logistic regression models. We included sex, age, and educational attainment as potential confounders in the multivariable model. We also performed stratified analysis by sex and geographic area (Southern versus Northern Italy, i.e., the Catania province as opposed to remaining provinces). We used Stata Software (v16.1, Stata Corp, College Station, TX, USA, 2019) for all data analysis.

## 3. Results

Table 1 shows the characteristics of the 230 participants who accepted to participate in the study. The average response rate was 19.0% (230/1211), higher in Emilia-Romagna (122/462 = 24.6%), followed by Piedmont (68/337 = 20.2%) and Sicily (40/412 = 9.7%).

Overall, we recruited 95 (men/women: 51/44) cases and 135 controls (men/women: 71/64). Mean age was 65.8 years (standard deviation: 10.9), slightly higher in cases. As regards educational attainment, a higher proportion of controls graduated at high school or higher than cases, while controls showed a lower proportion of unmarried and previously married participants. Five cases and two controls reported at least one relative within the second grade with an ALS diagnosis. Six cases revealed a repeat expansion mutation in the *C9orf72* gene, and two of them also reported an ALS diagnosis in the family. The majority of subjects were right-dominant handed and footed, while a few participants reported to be left-dominant or ambidextrous. The distribution of sites of ALS onset is reported in Table 2 according to handedness and footedness of cases. We found that right dominant handed cases have a slightly higher percentage of bulbar onset compared to non-right dominant handed. While we found no substantial difference in right or left-limb onset according to handedness, a higher percentage of contralateral onset for non-right dominant footed participants was noted. In addition, all cases with bulbar onset were right-dominant footed. When we divided sites according to lower or upper limb onset, we found a substantially comparable distribution across right and left handed/footed cases.

Table 3 shows the association between potential risk factors and ALS. In the analysis of dominant handedness and footedness, we found a positive association between ALS and both non-right dominant handed and footed subjects.

With a view to personal habits, we found an inverse association between risk and regular fish consumption (OR = 0.27, 95% CI 0.12–0.60). However, There was no evidence of a dose-response association because an intake equal to or higher than 3 times per week was required to show an OR of 0.58 (95% CI 0.22–1.58). Similarly, any wine consumption was inversely associated with ALS (OR = 0.46, 95% CI 0.26–0.82), whilst consumption of alcohol units higher than recommended (>2 alcohol units/day in men and >1/day unit in women) was not associated with disease risk. Use of private wells as a main source (≥70%) of drinking water showed a positive association compared with municipal tap water (OR = 1.44, 95% CI 0.29–7.03), along with the use of a private well as a source of drinking water (OR = 1.38, 95% CI 0.73–2.27). History of dietary supplements use in the past 20 years showed a virtually null association with the disease, although we found a higher ALS risk in the few subjects reporting use of amino acid supplements (OR = 2.81, 95% CI 0.47–16.87). Finally, while current smoking showed no association with ALS risk, history of ever smoking showed a positive though imprecise association (OR = 1.19, 95% CI 0.68–2.09).

As far as clinical history is concerned, a positive history of any trauma requesting medical evaluation showed a very loose association (OR = 1.14, 95% CI 0.64–2.01), but further stratified analysis demonstrated a positive association with head trauma (OR = 2.61, 95% CI 1.19–5.72), and partially with trunk trauma (OR = 1.76, 95% CI 0.64–4.85). Similarly, only head fracture seems positively associated with ALS, however based on its occurrence in 3 cases and 1 control only. We found no change in disease risk for a history of polio vaccine, blood donation, any surgery, including in those who had undergone general anesthesia. Finally, we found a higher risk for subjects reporting a positive history of ALS in the family (OR = 3.56, 95% CI 0.65–19.45).

We found a limited difference in risk for leisure activities (Table 4), alongside a positive association with use of herbicides during gardening, model making, and an inverse association with photograph darkroom printing. As for sports, playing any sport showed a slightly inverse association (OR = 0.77, 95% CI 0.41–1.42) including those at a competitive level. A slightly increased risk was associated with soccer at a competitive level (OR = 1.19, 95% IC 0.35–4.02), skiing (OR = 1.48, 95% CI 0.42–5.13), and marginally with swimming (OR = 1.61, 95% 0.09–27.33) due the very low number of exposed subjects. Sensitivity analysis after exclusion of subjects with a positive family history of ALS yielded substantially comparable results (Tables S1 and S2). Stratified analysis carried out in men and women separately showed substantially comparable results with some exceptions (Tables S3–S6). In particular, we found a higher risk for use of amalgam fillings in men but not in women, as well as for the use of private wells for drinking water and for use of selenium-containing supplements. Conversely, ever smoking posed a higher risk in women, along with the consumption of more alcohol units than recommended (Tables S3 and S4). In addition, we found higher estimates in men compared to women for any trauma, particularly head trauma, and any fracture. In relation to sports, a positive association was found with skiing in men, and with swimming in women, which was not evident from the overall analysis (Tables S5 and S6). Finally, in stratified analyses according to study area, i.e., Northern and Southern Italy, we noted a very high risk for ever smoking in the Southern but not in the Northern Italian population, as was the case for use of amalgam fillings (Tables S7 and S8). Similarly, use of private wells for drinking water was clearly associated with ALS risk in the Northern Italian population. On the other hand, we found a weaker association in the Southern Italian population, which in turn demonstrated a higher risk for use of private wells for irrigation (Tables S7 and S8). As concerns use of dietary supplements, interestingly, an increased risk emerged in the Catania population for the use of vitamin and mineral supplements, and for selenium-containing supplements. In addition, data indicated a higher risk for trauma, head trauma, and a history of general or spinal anesthesia, as compared to the Northern Italy population (Table S8). With regard to leisure activities, we found an increased risk for gardening and painting in the Catania population, while we found a positive association with ALS for pesticides and herbicides use for Northern Italy (Tables S9 and S10). We finally repeated all study analyses by excluding cases carrying the *C9orf72* gene mutation (Tables S11 and S12). No major change in strength and direction of relative risk estimates occurred in the analysis restricted to subjects without such gene mutation.

## 4. Discussion

Epidemiological investigations have suggested associations between ALS and a large number of environmental risk factors. However, evidence for each of them is still inadequate and inconsistent [21,22,23]. In this study, we investigated whether some of these potential environmental risk factors may play a role in the onset of sporadic ALS in four Italian populations from different areas of the country.

Interestingly, we observed an inverse association with fish consumption when we compared no consumption with regular consumption and used three or more fish-based meals as cutoff point. Nonetheless, no evidence of a dose-response relation emerged, and the association was considerably weaker in women than in men. A large cohort study carried out in the US investigating the role of dietary factors failed to confirm the association with fish intake [24], while another study carried out in Italy reported a decreased risk only in the highest quartile compared to the lowest [25]. A pooled analysis of five prospective US studies suggested a protective role for foods high in omega-6 and omega-3 long-chain polyunsaturated fatty acids [26]. These contrasting results may be due to the heterogeneity behind fish consumption, i.e., lake versus sea fish, kind of and relationship with possible contaminants [27].

We found a positive association between disease risk and use of private wells for drinking water but not for cultivation. This was the case despite the low number of exposed subjects and the fact that the association seems limited to men, which strongly reduces the reliability of our results. Previous studies have already suggested a positive association with the percentage of the population using well water and mortality rates for motor neuron diseases [28,29], possibly linked to fungal or bacterial contamination [30,31]. This mainly applies to cyanobacteria due to production of β-N-methylamino-L-alanine (BMAA), a neurotoxin that may enter the food chain [31,32,33,34]. Interestingly, other findings linked ALS with living near lakes with the presence of algal bloom [35,36]. However, we did not assess water contamination through sample analysis, thus hampering the possible link with a specific biological agent. It is noteworthy that a previous cohort study reported an increased risk of ALS associated with exposure to inorganic hexavalent selenium through municipal tap water [37,38]. In this study, however, we found that use of drinking water from municipal utilities is associated with lower ALS risk compared with the use of either municipal or bottled water. While selenium content of municipal tap water in Italy is usually negligible, its levels in bottled and particularly well water may vary considerably [39,40], and further investigation about the role of this or other elements in raising ALS risk through exposure via drinking water is definitely required [41]. Possible differences in well water contamination may also explain the stronger association we found for the Northern Italy population compared to the Southern one, a difference which warrants further investigation.

As regards alcohol intake, although our results indicated an inverse association between wine or alcohol consumption and ALS, consistent with other reports [25,42,43], null [44] and positive associations [26,45] have also been described. In addition, considering the adverse effects of alcohol, we carried out an analysis using recommended alcohol units as cutoff points. Overall, we found no difference in risk in participants, with a possible higher risk in women, suggesting caution in considering wine or alcohol to be either protective from risk factors for ALS.

The positive association we found between ALS and smoking habits is consistent with previous studies [42,45,46,47,48,49,50,51,52,53]. Despite a few studies reporting no difference or a decreasing risk in association with smoking [43,54,55], nevertheless, a detrimental effect of smoking has also been reported as a negative prognostic factor [56,57]. This strengths the relevance of quitting in either preventing the onset or delaying the progression of the disease.

As far as dominant handedness and site of onset are concerned, we found no concordance between dominant handedness and ipsilateral onset. Conversely, a higher percentage of cases signaled an upper-limb onset compared to lower limb onset. Our findings contrast with those from a previous study reporting a concordance between handedness and side of onset in upper limb onset patients [58]. Conversely, we found similar results in terms of absence of concordance between footedness and side of onset [58,59]. Potential reasons for such a difference in site of onset have been derived from the greater connectivity in the dominant motor cortex with respect to handedness [60]. This difference in excitability of dominant and non-dominant hemispheres [61] may be relevant to disease onset, due to the neuronal hyperexcitability occurring in the cortex during early stages of ALS [62,63]. A similar explanation was suggested for the higher risk of upper-limb as opposed to lower-limb onset.

With regard to dietary supplements, we found contrasting results. Although there were a low number of exposed subjects in such categories, only use of amino acid supplements showed a positive association with disease risk, while null/inverse relations could be noted for other supplements. It is noteworthy that a few investigations reported an inverse association with vitamin E supplements [64,65], but no significant associations were found for vitamin C or multivitamins use [65], consistent with our study. Interestingly, with reference to the positive association with use of amino acid compounds, an experimental study has shown that β-N-methylamino-L-alanine (BMAA), previously mentioned with regard to cyanobacteria bloom neurotoxicity [33], and the supplement β-alanine have similar and substantial comparable toxicity, despite their low levels [66]. In addition, a side effect of β-alanine supplementation is paresthesia (tingling) [67], suggesting caution in its administration, at least at high doses, within dietary supplements [68].

The hypothesis that trauma increases ALS risk has been suggested by several studies [43,69,70,71,72,73,74], with specific reference to head trauma [42,75,76,77,78,79,80], and column and trunk trauma [81,82]. However, inconsistent results have been reported by other studies [83,84,85,86]. One possible explanation for the increased ALS risk after traumatic events could be the inclusion of subjects as ALS cases who were affected by chronic traumatic encephalomyelopathy associated with motor neuron disease. This might have been confused with ALS, especially in older studies [13,87]. In our study, however, only ‘definite’ and ‘probable’ ALS cases were recruited, thus ruling out the occurrence of a misdiagnosis. Another possible explanation is that the enhanced occurrence of trauma may represent an early, subclinical sign of ALS onset, and may therefore be due to reverse causation [88]. However, in the analysis excluding head trauma over the previous five years from the date of diagnosis (*N* = 3 cases and 3 controls), we found comparable results (OR = 2.69, 95% CI 1.14–6.36).

In our study, we found a positive yet imprecise association between previous electric shocks and ALS. This confirms previous observations demonstrating an increased risk [84,89,90,91,92,93], but not others showing no association [94,95]. Electrical injury may cause a wide range of morphological changes in the central nervous system [96], including neuronophagia, neuronal chromatolysis, neuronal loss, and microglial activation, which is considered an early event in central nervous system damage [97].

Some medical procedures have been investigated in relation to ALS risk [43,82,89,98,99,100]. Since it has been suggested that viral infections may be associated with ALS [13,84,101], we investigated whether polio vaccination history could be associated with disease risk. However, we found null to inverse association, consistent with a previous study [99].

In our study, we found an imprecise inverse association with overall sport playing and ALS risk, and limited evidence of a positive association with competitive soccer. Competitive swimming also showed a positive association. Nonetheless, this was statistically very imprecise and weak, based as it was on two exposed subjects only, suggesting extreme caution in the interpretation of these results. Most studies evaluating overall and individual sport playing generally showed an increased risk [43,53,75,77,83,102,103,104,105,106,107], although different results have also been reported [42,54,94,108,109,110]. Remarkably, our results showed an increased risk for skiers, consistent with recent findings about Swiss competitive players [111]. In addition, a higher incidence of ALS has been reported in professional athletes, especially soccer players. Thus, an attempt has been made to understand the role of strenuous sport and physical activity [112,113,114]. Exercise could alter the extent of exposure or could influence the distribution, metabolism or potency of an excitotoxin [115]. It has been suggested that a physically active lifestyle and contact sports may be particularly associated with an increased probability of trauma [116], especially head trauma for some sports such as soccer or football [117]. As for other leisure activities, we find a loose association, in line with previous studies [54,95]. The only exception was a marginal increase for use of pesticides during gardening, particularly herbicides, which appears to be consistent with previous observations [54,118,119].

This study has some limitations that must be acknowledged. First of all, the population-based design of the study, particularly with reference to the eligible controls, was associated with a low response rate. This reduced the overall sample size and affected the precision of the risk estimates. As far as cases are concerned, we acknowledge that a few deceased and seriously ill subjects may have not been included. In such cases, we expected that the questionnaire could be filled out by caregivers or other relatives. Nevertheless, when we assessed the distribution of individual characteristics of study cases and controls, and we compared it with that of the general population [120,121], only limited differences emerged. This suggests that there is little evidence of selection bias. In particular, the distribution of educational level in controls was comparable to the Italian population over 35 years based on the 2011 census data (http://dati-censimentopopolazione.istat.it/ Index.aspx). The limited sample size is also due to the low ALS incidence in the study areas, around 1–2 cases/100,000 inhabitants [7,8,122,123].

We also acknowledge that the exposure assessment, solely based on self-report by the participants or their proxy respondents, could have been affected by imprecision due to the long period of time involved, and even by recall bias. This might have been the case especially when seeking long-term or historical information about former exposure to contaminants and other factors perceived by the cases as being associated with disease risk. In addition, the reliability and accuracy in recalling historical exposures dating back to several years before enrolment in the study may be challenged. However, we do not expect major differences between cases and controls in this matter, not even for differential recall bias due to the substantially limited awareness of potential risk factors for ALS. Finally, the observational design of the study does not allow us to rule out the possibility that some unmeasured confounding may have occurred.

Some strengths of our investigation should also be outlined. Firstly, it is worth pointing out its population-based design: we recruited controls from the general population, by randomly extracting them from Local Health Authority registries. This is mandatory for all Italian residents, thus limiting the occurrence of selection bias. Secondly, the study area encompassing four Italian provinces allowed us to increase the sample size of this case-control study, as compared with previous investigations. In addition, it enabled us to assess the associations under investigation in markedly different populations with reference to lifestyle and environmental exposures, if not genetic factors [124]. Finally, we were able to perform sensitivity analysis by excluding subjects reporting a history of ALS in their relatives or carrying the *C9orf72* genetic mutation [84], in order to assess the specific role of environmental risk factors for sporadic ALS. Such restricted analysis yielded no substantially different results.

## 5. Conclusions

The results of the present case-control study suggest a possible association between some non-genetic risk factors and ALS risk, namely trauma, particularly head trauma or fracture, electric shock, some competitive sport activities, use of private wells for drinking water, use of herbicides during gardening and consumption of some specific dietary supplements. Caution in interpreting these results should be used in light of the study limitations, such as the small sample size and the reduced statistical precision of the risk estimates, particularly when a low number of subjects are exposed, the potential for exposure misclassification and unmeasured confounding, and the risk of reverse causation due to the case-control study design. In addition, the toxicological mechanisms underpinning the association between the environmental factors identified in this study and disease risk remain to be elucidated. Therefore, further epidemiologic studies on the environmental determinants of ALS are clearly needed, and they should encompass a careful validation of historical exposures and control of potential confounders.

## Figures and Tables

**Table 1:** Characteristics of study population.

Characteristics	Cases *n* (%)	Controls *n* (%)	Total *n* (%)
All subjects	95 (100)	135 (100)	230 (100)
Sex			
Men	51 (53.7)	71 (52.6)	122 (53.0)
Women	44 (46.3)	64 (47.4)	108 (47.0)
Age			
Mean (SD) years	64.9 (11.7)	66.5 (10.3)	65.8 (10.9)
<65 years	46 (48.4)	56 (41.5)	102 (44.3)
≥65 years	49 (51.6)	79 (58.5)	128 (55.6)
Province of residence			
Catania	19 (20.0)	21 (15.6)	40 (17.4)
Modena	29 (30.5)	47 (34.8)	76 (33.0)
Reggio Emilia	13 (13.7)	33 (24.4)	46 (20.0)
Novara	34 (37.8)	34 (25.2)	68 (29.6)
Educational attainment			
Primary school or less	40 (42.1)	47 (34.8)	87 (37.8)
Middle school	24 (25.3)	28 (20.7)	52 (22.6)
High school	23 (24.2)	42 (31.1)	65 (28.3)
College or higher	8 (8.4)	18 (13.3)	26 (11.3)
Marital status			
Married	64 (67.4)	101 (74.8)	165 (71.7)
Unmarried	9 (9.5)	8 (5.9)	17 (7.4)
Previously married	22 (23.2)	26 (19.3)	48 (20.9)
Occupational sector			
Agriculture	9 (9.5)	8 (5.9)	17 (7.4)
Manufacturing	48 (50.5)	55 (40.7)	103 (44.8)
Services	38 (40.0)	72 (53.3)	110 (47.8)
Handedness			
Right dominant	83 (87.4)	124 (91.8)	207 (90.0)
Left dominant	4 (4.2)	4 (2.9)	8 (3.5)
Ambidextrous	8 (8.4)	7 (5.2)	15 (6.5)
Footedness			
Right dominant	83 (87.4)	123 (91.1)	206 (89.6)
Left dominant	11 (11.6)	10 (7.4)	21 (9.1)
Ambidextrous	1 (1.0)	2 (1.5)	3 (1.3)
ALS cases in the family			
Yes	5 (5.3)	2 (1.5)	7 (3.0)
No	90 (94.7)	133 (98.5)	233 (97.0)

ALS: Amyotrophic lateral sclerosis.

**Table 2:** Distribution of ALS onset sites according to handedness and footedness (divided into right and non-right dominant).

Type of Onset	Right Handed Dominant	Non-Right Handed Dominant	Right Footed Dominant	Non-Right Footed Dominant	Total *n* (%)
Laterality of onset					
Bilateral onset	16 (19.3)	3 (15.0)	16 (19.3)	3 (25.0)	19 (20.0)
Right-limb onset	26 (31.3)	4 (33.3)	24 (28.9)	6 (50.0)	30 (31.6)
Left-limb onset	10 (12.0)	2 (16.7)	11 (13.2)	1 (8.3)	12 (12.6)
Bulbar	25 (30.1)	2 (16.7)	27 (32.5)	0 (0.0)	27 (28.4)
Other/trunk	6 (7.2)	1 (8.3)	5 (6.2)	2 (16.7)	7 (7.4)
Upper/lower onset					
Upper limb onset	22 (26.5)	3 (25.0)	22 (26.5)	3 (25.0)	25 (26.3)
Right upper limb onset	15 (18.1)	2 (16.7)	15 (18.1)	2 (16.7)	17 (17.9)
Left upper limb onset	7 (8.4)	1 (8.3)	7 (8.4)	1 (8.3)	8 (8.4)
Lower limb onset	10 (12.0)	1 (8.3)	10 (12.0)	1 (8.3)	11 (11.6)
Right lower limb onset	8 (9.6)	0 (0.0)	7 (8.4)	0 (0.0)	7 (8.4)
Left lower limb onset	2 (2.4)	1 (8.3)	3 (2.4)	1 (8.3)	4 (4.2)
Bulbar	16 (19.3)	2 (16.7)	18 (21.7)	0 (0.0)	18 (18.9)
Mixed upper/lower right	3 (3.6)	1 (8.3)	2 (16.7)	2 (16.7)	4 (4.2)
Mixed upper/lower left	11 (13.2)	3 (25.0)	12 (14.5)	2 (16.7)	14 (14.7)
Other/trunk	21 (25.3)	2 (16.7)	19 (22.9)	4 (33.3)	23 (24.2)

**Table 3:** Odds ratio (OR) with 95% confidence interval (CI) of ALS risk according to personal characteristics and clinical factors.

Factors	Cases (y/*n*)	Controls (y/*n*)	OR ^a^	OR ^b^	(95% CI)
Personal information and habits					
Dominant hand					
Right-handed	83	124	Ref.		
Left-handed	4	4	1.49	1.45	(0.34–6.14)
Ambidextrous	8	7	1.71	1.73	(0.59–5.02)
Non-right handed	12/83	11/124	1.63	1.63	(0.67–3.92)
Dominant foot					
Right	85	123	Ref.		
Left	11	10	1.63	1.62	(0.64–4.06)
Ambidextrous	2	1	0.74	0.78	(0.07–8.84)
Non-right	12/83	12/123	1.48	1.47	(0.62–3.50)
Regular use of skin cream ^c^	25/43	66/68	0.60	0.53	(0.27–1.06)
Having or ever having had amalgam fillings ^c^	42/26	79/55	1.12	1.17	(0.62–2.20)
Regular use of chewing gum ^c^	16/52	18/116	1.99	2.09	(0.92–4.74)
Eat fish ^c^	48/20	120/14	0.28	0.27	(0.12–0.60)
Eat ≥3 fish-based meals per week ^c^	6/62	18/116	0.62	0.58	(0.22–1.58)
Wine drinking ^c^	43/52	85/50	0.49	0.46	(0.26–0.82)
Alcohol units intake ^c,d^	10/58	21/113	0.93	0.98	(0.42–2.28)
Main source of drinking water					
No preference	34	46	Ref.	Ref.	
Municipal water	24	55	0.59	0.55	(0.28–1.07)
Private wells	4	3	1.80	1.44	(0.29–7.03)
Bottled water	33	31	1.44	1.24	(0.62–2.44)
Current use of any private well water	12/83	9/126	2.02	1.91	(0.76–4.79)
Ever use a private well/fountain for drinking water	38/57	46/89	1.29	1.38	(0.73–2.27)
Current use of a private well/fountain for irrigation	14/81	30/105	0.60	0.57	(0.28–1.16)
Ever smoking	49/46	65/70	1.15	1.19	(0.68–2.09)
Current smoking	11/84	15/112	1.05	1.00	(0.42–2.35)
Use of the following dietary supplements in the former 20 years:	26/69	46/89	0.73	0.70	(0.39–1.25)
Vitamin supplements	17/78	29/106	0.80	0.80	(0.40–1.59)
Vitamin and mineral supplements	16/79	27/108	0.81	0.73	(0.36–1.47)
Aminoacidic supplements	4/91	2/133	2.92	2.81	(0.47–16.87)
Energy drinks	7/88	11/124	0.90	0.88	(0.31–2.51)
Selenium-containing supplements	23/72	37/98	0.85	0.88	(0.47–1.65)
Clinical history					
Any trauma requesting medical evaluation	35/60	49/86	1.02	1.14	(0.64–2.01)
Head trauma	19/76	13/122	2.35	2.61	(1.19–5.72)
Trunk trauma	9/86	8/127	1.66	1.76	(0.64–4.85)
Arm trauma	16/79	39/96	0.50	0.52	(0.27–1.02)
Any fracture	34/61	54/81	0.84	0.85	(0.49–1.48)
Head fracture	3/92	1/134	4.37	4.07	(0.40–41.30)
Trunk fracture	6/89	10/125	0.84	0.93	(0.31–2.73)
Arm fracture	26/69	45/90	0.75	0.75	(0.41–1.35)
Electric shock trauma	7/88	5/130	2.07	2.09	(0.62–7.06)
Previous Polio vaccine	41/54	66/69	0.79	0.75	(0.41–1.35)
Previous spinal anesthesia	24/71	36/99	0.93	0.94	(0.51–1.74)
Ever been blood donor	24/71	37/98	0.90	0.98	(0.53–1.84)
Any surgery	76/19	113/22	0.78	0.75	(0.37–1.53)
with general anesthesia	23/53	35/78	0.97	0.97	(0.51–1.85)
ALS cases in the family	5/90	2/133	3.69	3.56	(0.65–19.45)

^a^ Crude model; ^b^ Model adjusted by sex, age, and educational attainment; ^c^ Section missing for 28 subjects due to pilot version of the questionnaire. Analysis performed in 202 participants only (68 cases/134 controls); ^d^ Recommended alcohol units (two alcohol units in men, one unit in women) used as cutoff points.

**Table 4:** Odds ratio (OR) with 95% confidence interval (CI) of ALS risk according to leisure activities and other lifestyle factors.

Factor	Cases (y/*n*)	Controls (y/*n*)	OR ^a^	OR ^b^	(95% CI)
Hunting	4/91	6/129	0.95	1.05	(0.27–4.09)
Fishing	20/75	37/98	0.71	0.60	(0.29–1.24)
Using lead	16/79	32/103	0.65	0.57	(0.27–1.22)
Using lead in fishermen only	16/4	32/5	0.62	0.86	(0.15–4.75)
Painting	6/89	13/122	0.63	0.70	(0.25–1.98)
Use of oil paints	2/93	8/127	0.34	0.39	(0.08–1.96)
Use of oil paints in painters only	2/4	8/5	0.31	0.43	(0.04–4.90)
Model-making	7/88	8/127	1.26	1.39	(0.47–4.15)
Gardening	42/53	65/70	0.85	0.95	(0.55–1.64)
Any use of pesticides?	20/75	28/107	1.02	1.06	(0.55–2.05)
Using pesticides for gardening?	16/79	21/114	1.10	1.15	(0.56–2.39)
Using herbicides for gardening?	16/79	13/122	1.90	1.95	(0.88–4.36)
Using fungicides for gardening?	7/88	12/123	0.82	0.93	(0.35–2.51)
Photograph darkroom printing?	5/90	24/111	0.26	0.24	(0.09–0.70)
Play Sports	38/57	63/72	0.76	0.77	(0.41–1.42)
Competitive sports	10/85	22/113	0.60	0.50	(0.22–1.17)
Soccer	14/81	21/114	0.94	0.75	(0.33–1.72)
Competitive soccer	6/89	6/129	1.45	1.19	(0.35–4.02)
Volleyball	5/90	11/124	0.63	0.59	(0.19–1.87)
Competitive volleyball	1/94	4/131	0.35	0.28	(0.03–2.64)
Cycling	5/90	7/128	1.02	1.00	(0.29–3.44)
Competitive cycling	1/94	5/131	0.35	0.31	(0.03–2.94)
Swimming	5/90	7/128	1.02	1.12	(0.31–4.06)
Competitive swimming	1/94	1/134	1.43	1.61	(0.09–27.33)
Skiing	5/90	7/128	1.02	1.48	(0.42–5.13)
Competitive skiing	0/95	0/135	-	-	
Athletics	7/88	16/119	0.59	0.66	(0.25–1.74)
Competitive athletics	0/95	6/129	-	-	
Tennis	4/94	7/128	0.80	0.96	(0.26–3.53)
Competitive tennis	1/94	0/135	-	-	

^a^ Crude model; ^b^ Model adjusted by sex, age, and educational attainment.

## Supplementary Materials

The following are available online at https://www.mdpi.com/1660-4601/17/3/857/s1, Table S1. Odds ratio (OR) with 95% confidence interval (CI) of ALS risk according to personal characteristics and clinical factors without subjects with a family history of ALS. Table S2. Odds ratio (OR) with 95% confidence interval (CI) of ALS risk according to leisure activities and other lifestyle factors without subjects with a family history of ALS. Table S3. Odds ratio (OR) with 95% confidence interval (CI) of ALS risk according to personal characteristics and clinical factors in men. Table S4. Odds ratio (OR) with 95% confidence interval (CI) of ALS risk according to personal characteristics and clinical factors in women. Table S5. Odds ratio (OR) with 95% confidence interval (CI) of ALS risk according to leisure activities and other lifestyle factors in men. Table S6. Odds ratio (OR) with 95% confidence interval (CI) of ALS risk according to leisure activities and other lifestyle factors in women. Table S7. Odds ratio (OR) with 95% confidence interval (CI) of ALS risk according to personal characteristics and clinical factors in the Northern Italy provinces of Modena, Novara and Reggio Emilia. Table S8. Odds ratio (OR) with 95% confidence interval (CI) of ALS risk according to personal characteristics and clinical factors in the Southern Italy province of Catania. Table S9. Odds ratio (OR) with 95% confidence interval (CI) of ALS risk according to leisure activities and other lifestyle factors in the Northern Italy provinces of Modena, Novara and Reggio Emilia. Table S10. Odds ratio (OR) with 95% confidence interval (CI) of ALS risk according to leisure activities and other lifestyle factors in the Southern Italy province of Catania. Table S11. Odds ratio (OR) with 95% confidence interval (CI) of ALS risk according to personal characteristics and clinical factors without carriers of *C9orf72* mutation. Table S12. Odds ratio (OR) with 95% confidence interval (CI) of ALS risk according to leisure activities and other lifestyle factors without carriers of *C9orf72* mutation.

## References

[1] Gordon P.H. (2013). Amyotrophic lateral sclerosis: An update for 2013 clinical features, pathophysiology, management and therapeutic trials. Aging Dis..

[2] Valadi N. (2015). Evaluation and management of amyotrophic lateral sclerosis. Prim. Care.

[3] Gale C.R., Braidwood E.A., Winter P.D., Martyn C.N. (1999). Mortality from Parkinson’s disease and other causes in men who were prisoners of war in the far east. Lancet.

[4] Talbott E.O., Malek A.M., Lacomis D. (2016). The epidemiology of amyotrophic lateral sclerosis. Handb. Clin. Neurol..

[5] Marin B., Boumediene F., Logroscino G., Couratier P., Babron M.C., Leutenegger A.L., Copetti M., Preux P.M., Beghi E. (2017). Variation in worldwide incidence of amyotrophic lateral sclerosis: A meta-analysis. Int. J. Epidemiol..

[6] Hardiman O., Al-Chalabi A., Brayne C., Beghi E., van den Berg L.H., Chiò A., Martin S., Logroscino G., Rooney J. (2017). The changing picture of amyotrophic lateral sclerosis: Lessons from European registers. J. Neurol. Neurosurg. Psychiatry.

[7] Tesauro M., Consonni M., Filippini T., Mazzini L., Pisano F., Chiò A., Esposito A., Vinceti M. (2017). Incidence of amyotrophic lateral sclerosis in the province of Novara, Italy, and possible role of environmental pollution. Amyotroph. Lateral Scler. Front. Degener..

[8] Mandrioli J., Biguzzi S., Guidi C., Venturini E., Sette E., Terlizzi E., Ravasio A., Casmiro M., Salvi F., Liguori R. (2014). Epidemiology of amyotrophic lateral sclerosis in Emilia Romagna region (Italy): A population based study. Amyotroph. Lateral Scler. Front. Degener..

[9] Nicoletti A., Vasta R., Venti V., Mostile G., Lo Fermo S., Patti F., Scillieri R., De Cicco D., Volanti P., Marziolo R. (2016). The epidemiology of amyotrophic lateral sclerosis in the Mount Etna region: A possible pathogenic role of volcanogenic metals. Eur. J. Neurol..

[10] Chiò A., Mora G., Moglia C., Manera U., Canosa A., Cammarosano S., Ilardi A., Bertuzzo D., Bersano E., Cugnasco P. (2017). Secular trends of amyotrophic lateral sclerosis: The Piemonte and Valle d’Aosta register. JAMA Neurol..

[11] Boumediene F., Vasta R., Rascuna C., Lo Fermo S., Volanti P., Marziolo R., Patti F., Ferrante M., Preux P.M., Marin B. (2019). Amyotrophic lateral sclerosis spatial epidemiology in the Mount Etna region, Italy. Eur. J. Neurol..

[12] Zufiria M., Gil-Bea F.J., Fernandez-Torron R., Poza J.J., Munoz-Blanco J.L., Rojas-Garcia R., Riancho J., de Munain A.L. (2016). ALS: A bucket of genes, environment, metabolism and unknown ingredients. Prog. Neurobiol..

[13] Ingre C., Roos P.M., Piehl F., Kamel F., Fang F. (2015). Risk factors for amyotrophic lateral sclerosis. Clin. Epidemiol..

[14] Bozzoni V., Pansarasa O., Diamanti L., Nosari G., Cereda C., Ceroni M. (2016). Amyotrophic lateral sclerosis and environmental factors. Funct. Neurol..

[15] Oskarsson B., Horton D.K., Mitsumoto H. (2015). Potential environmental factors in amyotrophic lateral sclerosis. Neurol. Clin..

[16] Vinceti M., Bottecchi I., Fan A., Finkelstein Y., Mandrioli J. (2012). Are environmental exposures to selenium, heavy metals, and pesticides risk factors for amyotrophic lateral sclerosis?. Rev. Environ. Health.

[17] Vinceti M., Violi F., Tzatzarakis M., Mandrioli J., Malagoli C., Hatch E.E., Fini N., Fasano A., Rakitskii V.N., Kalantzi O.I. (2017). Pesticides, polychlorinated biphenyls and polycyclic aromatic hydrocarbons in cerebrospinal fluid of amyotrophic lateral sclerosis patients: A case-control study. Environ. Res..

[18] Vinceti M., Filippini T., Mandrioli J., Violi F., Bargellini A., Weuve J., Fini N., Grill P., Michalke B. (2017). Lead, cadmium and mercury in cerebrospinal fluid and risk of amyotrophic lateral sclerosis: A case-control study. J. Trace Elem. Med. Biol.

[19] Vinceti M., Filippini T., Wise L.A. (2018). Environmental selenium and human health: An update. Curr. Environ. Health Rep..

[20] Brooks B.R., Miller R.G., Swash M., Munsat T.L., Amyotrophic Lateral Sclerosis and Other Motor Neuron Disorders (2000). El Escorial revisited: Revised criteria for the diagnosis of amyotrophic lateral sclerosis. Amyotroph. Lateral Scler Other Motor Neuron Disord..

[21] Vinceti M. (2012). The environment and amyotrophic lateral sclerosis: Converging clues from epidemiologic studies worldwide. N. Am. J. Med. Sci..

[22] Belbasis L., Bellou V., Evangelou E. (2016). Environmental risk factors and amyotrophic lateral sclerosis: An umbrella review and critical assessment of current evidence from systematic reviews and meta-analyses of observational studies. Neuroepidemiology.

[23] Martin S., Al Khleifat A., Al-Chalabi A. (2017). What causes amyotrophic lateral sclerosis?. F1000Res.

[24] Morozova N., Weisskopf M.G., McCullough M.L., Munger K.L., Calle E.E., Thun M.J., Ascherio A. (2008). Diet and amyotrophic lateral sclerosis. Epidemiology.

[25] Pupillo E., Bianchi E., Chio A., Casale F., Zecca C., Tortelli R., Beghi E. (2018). Amyotrophic lateral sclerosis and food intake. Amyotroph. Lateral Scler. Front. Degener..

[26] Fitzgerald K.C., O’Reilly E.J., Falcone G.J., McCullough M.L., Park Y., Kolonel L.N., Ascherio A. (2014). Dietary omega-3 polyunsaturated fatty acid intake and risk for amyotrophic lateral sclerosis. JAMA Neurol..

[27] Lance E., Arnich N., Maignien T., Bire R. (2018). Occurrence of beta-n-methylamino-l-alanine (BMAA) and isomers in aquatic environments and aquatic food sources for humans. Toxins.

[28] Schwartz G.G., Klug M.G. (2016). Motor neuron disease mortality rates in U.S. States are associated with well water use. Amyotroph. Lateral Scler. Front. Degener..

[29] Schwartz G.G., Rundquist B.C., Simon I.J., Swartz S.E. (2017). Geographic distributions of motor neuron disease mortality and well water use in U.S. Counties. Amyotroph. Lateral Scler. Front. Degener..

[30] French P.W., Ludowyke R.I., Guillemin G.J. (2019). Fungal-contaminated grass and well water and sporadic amyotrophic lateral sclerosis. Neural Regen. Res..

[31] Stipa G., Manganelli M., Lolli F. (2013). Cyanobacteria biomagnification and amyotrophic lateral sclerosis. Med. Hypotheses.

[32] Buratti F.M., Manganelli M., Vichi S., Stefanelli M., Scardala S., Testai E., Funari E. (2017). Cyanotoxins: Producing organisms, occurrence, toxicity, mechanism of action and human health toxicological risk evaluation. Arch. Toxicol..

[33] Bradley W.G., Mash D.C. (2009). Beyond Guam: The cyanobacteria/BMAA hypothesis of the cause of ALS and other neurodegenerative diseases. Amyotroph. Lateral Scler..

[34] Spencer P.S., Ludolph A.C., Kisby G.E. (1993). Neurologic diseases associated with use of plant components with toxic potential. Environ. Res..

[35] Torbick N., Hession S., Stommel E., Caller T. (2014). Mapping amyotrophic lateral sclerosis lake risk factors across northern New England. Int. J. Health Geogr..

[36] Caller T.A., Doolin J.W., Haney J.F., Murby A.J., West K.G., Farrar H.E., Ball A., Harris B.T., Stommel E.W. (2009). A cluster of amyotrophic lateral sclerosis in New Hampshire: A possible role for toxic cyanobacteria blooms. Amyotroph. Lateral Scler..

[37] Vinceti M., Filippini T., Malagoli C., Violi F., Mandrioli J., Consonni D., Rothman K.J., Wise L.A. (2019). Amyotrophic lateral sclerosis incidence following exposure to inorganic selenium in drinking water: A long-term follow-up. Environ. Res..

[38] Vinceti M., Ballotari P., Steinmaus C., Malagoli C., Luberto F., Malavolti M., Rossi P.G. (2016). Long-term mortality patterns in a residential cohort exposed to inorganic selenium in drinking water. Environ. Res..

[39] Vinceti M., Crespi C.M., Bonvicini F., Malagoli C., Ferrante M., Marmiroli S., Stranges S. (2013). The need for a reassessment of the safe upper limit of selenium in drinking water. Sci. Total Environ..

[40] Vinceti M., Bonvicini F., Rothman K.J., Vescovi L., Wang F. (2010). The relation between amyotrophic lateral sclerosis and inorganic selenium in drinking water: A population-based case-control study. Environ. Health.

[41] Vinceti M., Filippini T., Cilloni S., Bargellini A., Vergoni A.V., Tsatsakis A., Ferrante M. (2017). Health risk assessment of environmental selenium: Emerging evidence and challenges (review). Mol. Med. Rep..

[42] Lian L., Liu M., Cui L., Guan Y., Liu T., Cui B., Zhang K., Tai H., Shen D. (2019). Environmental risk factors and amyotrophic lateral sclerosis (ALS): A case-control study of als in china. J. Clin. Neurosci. Off. J. Neurosurg. Soc. Australas..

[43] Savettieri G., Salemi G., Arcara A., Cassata M., Castiglione M.G., Fierro B. (1991). A case-control study of amyotrophic lateral sclerosis. Neuroepidemiology.

[44] D’Ovidio F., Rooney J.P.K., Visser A.E., Manera U., Beghi E., Logroscino G., Vermeulen R.C.H., Veldink J.H., van den Berg L.H., Hardiman O. (2019). Association between alcohol exposure and the risk of amyotrophic lateral sclerosis in the euro-motor study. J. Neurol. Neurosurg Psychiatry.

[45] Kamel F., Umbach D.M., Munsat T.L., Shefner J.M., Sandler D.P. (1999). Association of cigarette smoking with amyotrophic lateral sclerosis. Neuroepidemiology.

[46] Armon C. (2009). Smoking may be considered an established risk factor for sporadic ALS. Neurology.

[47] Fang F., Bellocco R., Hernan M.A., Ye W. (2006). Smoking, snuff dipping and the risk of amyotrophic lateral sclerosis—A prospective cohort study. Neuroepidemiology.

[48] Gallo V., Bueno-De-Mesquita H.B., Vermeulen R., Andersen P.M., Kyrozis A., Linseisen J., Kaaks R., Allen N.E., Roddam A.W., Boshuizen H.C. (2009). Smoking and risk for amyotrophic lateral sclerosis: Analysis of the epic cohort. Ann. Neurol..

[49] Wang H., O’Reilly E.J., Weisskopf M.G., Logroscino G., McCullough M.L., Thun M.J., Schatzkin A., Kolonel L.N., Ascherio A. (2011). Smoking and risk of amyotrophic lateral sclerosis: A pooled analysis of 5 prospective cohorts. Arch. Neurol..

[50] Weisskopf M.G., McCullough M.L., Calle E.E., Thun M.J., Cudkowicz M., Ascherio A. (2004). Prospective study of cigarette smoking and amyotrophic lateral sclerosis. Am. J. Epidemiol..

[51] de Jong S.W., Huisman M.H., Sutedja N.A., van der Kooi A.J., de Visser M., Schelhaas H.J., Fischer K., Veldink J.H., van den Berg L.H. (2012). Smoking, alcohol consumption, and the risk of amyotrophic lateral sclerosis: A population-based study. Am. J. Epidemiol..

[52] Sutedja N.A., Veldink J.H., Fischer K., Kromhout H., Wokke J.H., Huisman M.H., Heederik D.J., Van den Berg L.H. (2007). Lifetime occupation, education, smoking, and risk of ALS. Neurology.

[53] Veldink J.H., Kalmijn S., Groeneveld G.J., Titulaer M.J., Wokke J.H., van den Berg L.H. (2005). Physical activity and the association with sporadic ALS. Neurology.

[54] Yu Y., Su F.C., Callaghan B.C., Goutman S.A., Batterman S.A., Feldman E.L. (2014). Environmental risk factors and amyotrophic lateral sclerosis (ALS): A case-control study of ALS in Michigan. PLoS ONE.

[55] Pamphlett R., Ward E.C. (2012). Smoking is not a risk factor for sporadic amyotrophic lateral sclerosis in an Australian population. Neuroepidemiology.

[56] Calvo A., Canosa A., Bertuzzo D., Cugnasco P., Solero L., Clerico M., De Mercanti S., Bersano E., Cammarosano S., Ilardi A. (2016). Influence of cigarette smoking on ALS outcome: A population-based study. J. Neurol. Neurosurg Psychiatry.

[57] Wood H. (2016). Motor neuron disease: Smoking adversely affects survival in patients with amyotrophic lateral sclerosis. Nat. Rev. Neurol..

[58] Turner M.R., Wicks P., Brownstein C.A., Massagli M.P., Toronjo M., Talbot K., Al-Chalabi A. (2011). Concordance between site of onset and limb dominance in amyotrophic lateral sclerosis. J. Neurol. Neurosurg Psychiatry.

[59] Devine M.S., Woodhouse H., McCombe P.A., Henderson R.D. (2013). The relationship between limb dominance, disease lateralization and spread of weakness in amyotrophic lateral sclerosis (ALS). Amyotroph. Lateral Scler. Front. Degener..

[60] Hammond G. (2002). Correlates of human handedness in primary motor cortex: A review and hypothesis. Neurosci. Biobehav. Rev..

[61] Baumer T., Dammann E., Bock F., Kloppel S., Siebner H.R., Munchau A. (2007). Laterality of interhemispheric inhibition depends on handedness. Exp. Brain Res..

[62] Rothstein J.D. (2009). Current hypotheses for the underlying biology of amyotrophic lateral sclerosis. Ann. Neurol..

[63] Bae J.S., Simon N.G., Menon P., Vucic S., Kiernan M.C. (2013). The puzzling case of hyperexcitability in amyotrophic lateral sclerosis. J. Clin. Neurol..

[64] Wang H., O’Reilly E.J., Weisskopf M.G., Logroscino G., McCullough M.L., Schatzkin A., Kolonel L.N., Ascherio A. (2011). Vitamin e intake and risk of amyotrophic lateral sclerosis: A pooled analysis of data from 5 prospective cohort studies. Am. J. Epidemiol..

[65] Ascherio A., Weisskopf M.G., O’Reilly E J., Jacobs E.J., McCullough M.L., Calle E.E., Cudkowicz M., Thun M.J. (2005). Vitamin e intake and risk of amyotrophic lateral sclerosis. Ann. Neurol..

[66] Lee M., McGeer P.L. (2012). Weak bmaa toxicity compares with that of the dietary supplement beta-alanine. Neurobiol. Aging.

[67] Trexler E.T., Smith-Ryan A.E., Stout J.R., Hoffman J.R., Wilborn C.D., Sale C., Kreider R.B., Jager R., Earnest C.P., Bannock L. (2015). International society of sports nutrition position stand: Beta-alanine. J. Int. Soc. Sports Nutr..

[68] Manuel M., Heckman C.J. (2011). Stronger is not always better: Could a bodybuilding dietary supplement lead to ALS?. Exp. Neurol..

[69] Kurland L.T., Radhakrishnan K., Smith G.E., Armon C., Nemetz P.N. (1992). Mechanical trauma as a risk factor in classic amyotrophic lateral sclerosis: Lack of epidemiologic evidence. J. Neurol. Sci..

[70] Peters T.L., Weibull C.E., Fang F., Sandler D.P., Lambert P.C., Ye W., Kamel F. (2017). Association of fractures with the incidence of amyotrophic lateral sclerosis. Amyotroph. Lateral Scler. Front. Degener..

[71] Gallagher J.P., Sanders M. (1987). Trauma and amyotrophic lateral sclerosis: A report of 78 patients. Acta Neurol. Scand..

[72] Wang M.D., Little J., Gomes J., Cashman N.R., Krewski D. (2016). Identification of risk factors associated with onset and progression of amyotrophic lateral sclerosis using systematic review and meta-analysis. Neurotoxicology.

[73] Riggs J.E. (1996). Amyotrophic lateral sclerosis, heterogeneous susceptibility, trauma, and epidemiology. Arch. Neurol..

[74] Sienko D.G., Davis J.P., Taylor J.A., Brooks B.R. (1990). Amyotrophic lateral sclerosis. A case-control study following detection of a cluster in a small Wisconsin community. Arch. Neurol..

[75] Strickland D., Smith S.A., Dolliff G., Goldman L., Roelofs R.I. (1996). Physical activity, trauma, and ALS: A case-control study. Acta Neurol. Scand..

[76] Williams D.B., Annegers J.F., Kokmen E., O’Brien P.C., Kurland L.T. (1991). Brain injury and neurologic sequelae: A cohort study of dementia, parkinsonism, and amyotrophic lateral sclerosis. Neurology.

[77] Halperin Ben Ami T., Riccò M., Odone A., Boccumi C., Vinceti M., Pietrini V., Signorelli C. (2010). Sclérose latérale amyotrophique: Analyse des facteurs de risques professionnels. Arch. Mal. Prof. L’Environnemen.

[78] Chen H., Richard M., Sandler D.P., Umbach D.M., Kamel F. (2007). Head injury and amyotrophic lateral sclerosis. Am. J. Epidemiol..

[79] Tsai C.P., Hu C., Lee C.T. (2019). Finding diseases associated with amyotrophic lateral sclerosis: A total population-based case-control study. Amyotroph. Lateral Scler. Front. Degener..

[80] Seelen M., van Doormaal P.T., Visser A.E., Huisman M.H., Roozekrans M.H., de Jong S.W., van der Kooi A.J., de Visser M., Voermans N.C., Veldink J.H. (2014). Prior medical conditions and the risk of amyotrophic lateral sclerosis. J. Neurol..

[81] Bruske-Hohlfeld I., Merritt J.L., Onofrio B.M., Stonnington H.H., Offord K.P., Bergstralh E.J., Beard C.M., Melton L.J., Kurland L.T. (1990). Incidence of lumbar disc surgery. A population-based study in Olmsted county, Minnesota, 1950–1979. Spine.

[82] Chio A., Meineri P., Tribolo A., Schiffer D. (1991). Risk factors in motor neuron disease: A case-control study. Neuroepidemiology.

[83] Beghi E., Logroscino G., Chiò A., Hardiman O., Millul A., Mitchell D., Swingler R., Traynor B.J. (2010). Amyotrophic lateral sclerosis, physical exercise, trauma and sports: Results of a population-based pilot case-control study. Amyotroph. Lateral Scler..

[84] Cruz D.C., Nelson L.M., McGuire V., Longstreth W.T. (1999). Physical trauma and family history of neurodegenerative diseases in amyotrophic lateral sclerosis: A population-based case-control study. Neuroepidemiology.

[85] Armon C. (2007). Sports and trauma in amyotrophic lateral sclerosis revisited. J. Neurol. Sci..

[86] Valenti M., Pontieri F.E., Conti F., Altobelli E., Manzoni T., Frati L. (2005). Amyotrophic lateral sclerosis and sports: A case-control study. Eur. J. Neurol..

[87] Factor-Litvak P., Al-Chalabi A., Ascherio A., Bradley W., Chiò A., Garruto R., Hardiman O., Kamel F., Kasarskis E., McKee A. (2013). Current pathways for epidemiological research in amyotrophic lateral sclerosis. Amyotroph. Lateral Scler. Front. Degener..

[88] Watanabe Y., Watanabe T. (2017). Meta-analytic evaluation of the association between head injury and risk of amyotrophic lateral sclerosis. Eur. J. Epidemiol..

[89] Deapen D.M., Henderson B.E. (1986). A case-control study of amyotrophic lateral sclerosis. Am. J. Epidemiol..

[90] Fischer H., Kheifets L., Huss A., Peters T.L., Vermeulen R., Ye W., Fang F., Wiebert P., Vergara X.P., Feychting M. (2015). Occupational exposure to electric shocks and magnetic fields and amyotrophic lateral sclerosis in Sweden. Epidemiology.

[91] Huss A., Spoerri A., Egger M., Kromhout H., Vermeulen R., Swiss National Cohort (2015). Occupational exposure to magnetic fields and electric shocks and risk of ALS: The Swiss National Cohort. Amyotroph. Lateral Scler. Front. Degener..

[92] Johansen C., Olsen J.H. (1998). Mortality from amyotrophic lateral sclerosis, other chronic disorders, and electric shocks among utility workers. Am. J. Epidemiol..

[93] Vergara X., Mezei G., Kheifets L. (2015). Case-control study of occupational exposure to electric shocks and magnetic fields and mortality from amyotrophic lateral sclerosis in the US, 1991–1999. J. Expo. Sci. Environ. Epidemiol..

[94] Gunnarsson L.G., Bodin L., Soderfeldt B., Axelson O. (1992). A case-control study of motor neurone disease: Its relation to heritability, and occupational exposures, particularly to solvents. Br. J. Ind. Med..

[95] Chancellor A.M., Slattery J.M., Fraser H., Warlow C.P. (1993). Risk factors for motor neuron disease: A case-control study based on patients from the Scottish Motor Neuron Disease Register. J. Neurol. Neurosurg Psychiatry.

[96] Abhinav K., Al-Chalabi A., Hortobagyi T., Leigh P.N. (2007). Electrical injury and amyotrophic lateral sclerosis: A systematic review of the literature. J. Neurol. Neurosurg Psychiatry.

[97] Kreutzberg G.W. (1996). Microglia: A sensor for pathological events in the cns. Trends Neurosci..

[98] Kondo K., Tsubaki T. (1981). Case-control studies of motor neuron disease: Association with mechanical injuries. Arch. Neurol..

[99] Mitchell J.D., Davies R.B., al-Hamad A., Gatrell A.C., Batterby G. (1995). MND risk factors: An epidemiological study in the north west of England. J. Neurol. Sci..

[100] Gresham L.S., Molgaard C.A., Golbeck A.L., Smith R. (1986). Amyotrophic lateral sclerosis and occupational heavy metal exposure: A case-control study. Neuroepidemiology.

[101] Cermelli C., Vinceti M., Beretti F., Pietrini V., Nacci G., Pietrosemoli P., Bartoletti A., Guidetti D., Sola P., Bergomi M. (2003). Risk of sporadic amyotrophic lateral sclerosis associated with seropositivity for herpesviruses and echovirus-7. Eur. J. Epidemiol..

[102] Scarmeas N., Shih T., Stern Y., Ottman R., Rowland L.P. (2002). Premorbid weight, body mass, and varsity athletics in ALS. Neurology.

[103] Harwood C.A., Westgate K., Gunstone S., Brage S., Wareham N.J., McDermott C.J., Shaw P.J. (2016). Long-term physical activity: An exogenous risk factor for sporadic amyotrophic lateral sclerosis?. Amyotroph. Lateral Scler. Front. Degener..

[104] Huisman M.H., Seelen M., de Jong S.W., Dorresteijn K.R., van Doormaal P.T., van der Kooi A.J., de Visser M., Schelhaas H.J., van den Berg L.H., Veldink J.H. (2013). Lifetime physical activity and the risk of amyotrophic lateral sclerosis. J. Neurol. Neurosurg Psychiatry.

[105] Okamoto K., Kihira T., Kondo T., Kobashi G., Washio M., Sasaki S., Yokoyama T., Miyake Y., Sakamoto N., Inaba Y. (2009). Lifestyle factors and risk of amyotrophic lateral sclerosis: A case-control study in Japan. Ann. Epidemiol..

[106] Belli S., Vanacore N. (2005). Proportionate mortality of italian soccer players: Is amyotrophic lateral sclerosis an occupational disease?. Eur. J. Epidemiol..

[107] Taioli E. (2007). All causes of mortality in male professional soccer players. Eur. J. Public Health.

[108] Gallo V., Vanacore N., Bueno-de-Mesquita H.B., Vermeulen R., Brayne C., Pearce N., Wark P.A., Ward H.A., Ferrari P., Jenab M. (2016). Physical activity and risk of amyotrophic lateral sclerosis in a prospective cohort study. Eur. J. Epidemiol..

[109] Pupillo E., Messina P., Giussani G., Logroscino G., Zoccolella S., Chiò A., Calvo A., Corbo M., Lunetta C., Marin B. (2014). Physical activity and amyotrophic lateral sclerosis: A European population-based case-control study. Ann. Neurol..

[110] Longstreth W.T., McGuire V., Koepsell T.D., Wang Y., van Belle G. (1998). Risk of amyotrophic lateral sclerosis and history of physical activity: A population-based case-control study. Arch. Neurol..

[111] Fang F., Hallmarker U., James S., Ingre C., Michaelsson K., Ahlbom A., Feychting M. (2016). Amyotrophic lateral sclerosis among cross-country skiers in Sweden. Eur. J. Epidemiol..

[112] Chiò A., Benzi G., Dossena M., Mutani R., Mora G. (2005). Severely increased risk of amyotrophic lateral sclerosis among Italian professional football players. Brain J. Neurol..

[113] Chiò A., Calvo A., Dossena M., Ghiglione P., Mutani R., Mora G. (2009). ALS in Italian professional soccer players: The risk is still present and could be soccer-specific. Amyotroph. Lateral Scler..

[114] Lacorte E., Ferrigno L., Leoncini E., Corbo M., Boccia S., Vanacore N. (2016). Physical activity, and physical activity related to sports, leisure and occupational activity as risk factors for ALS: A systematic review. Neurosci. Biobehav. Rev..

[115] Longstreth W.T., Nelson L.M., Koepsell T.D., van Belle G. (1991). Hypotheses to explain the association between vigorous physical activity and amyotrophic lateral sclerosis. Med. Hypotheses.

[116] Blecher R., Elliott M.A., Yilmaz E., Dettori J.R., Oskouian R.J., Patel A., Clarke A., Hutton M., McGuire R., Dunn R. (2019). Contact sports as a risk factor for amyotrophic lateral sclerosis: A systematic review. Global Spine J..

[117] Feddermann-Demont N., Junge A., Weber K.P., Weller M., Dvorak J., Tarnutzer A.A. (2017). Prevalence of potential sports-associated risk factors in Swiss amyotrophic lateral sclerosis patients. Brain Behav..

[118] Morahan J.M., Pamphlett R. (2006). Amyotrophic lateral sclerosis and exposure to environmental toxins: An Australian case-control study. Neuroepidemiology.

[119] Pamphlett R. (2012). Exposure to environmental toxins and the risk of sporadic motor neuron disease: An expanded Australian case-control study. Eur. J. Neurol..

[120] Ferrante G., Minardi V., Possenti V., Quarchioni E., Masocco M., Salmaso S., Braggion M., Campostrini S., Baldissera S., Gruppo Tecnico PASSI (2012). [Smoke: Prevalence is decreasing, but the gap between socioeconomic categories remains]. Epidemiol. Prev..

[121] Gruppo Tecnico PASSI Emilia-Romagna Sovrappeso e obesità in Emilia-Romagna: Dati del sistema di sorveglianza PASSI (anni 2008-2011). https://www.epicentro.iss.it/passi/pdf2012/EccessoPond_PassiER_08_11.pdf.

[122] Ragonese P., Cellura E., Aridon P., D’Amelio M., Spataro R., Taiello A.C., Maimone D., La Bella V., Savettieri G. (2012). Incidence of amyotrophic lateral sclerosis in Sicily: A population based study. Amyotroph. Lateral Scler..

[123] Bonvicini F., Vinceti M., Marcello N., Rodolfi R., Rinaldi M. (2008). The epidemiology of amyotrophic lateral sclerosis in Reggio Emilia, Italy. Amyotroph. Lateral Scler..

[124] Vinceti M., Fiore M., Signorelli C., Odone A., Tesauro M., Consonni M., Arcolin E., Malagoli C., Mandrioli J., Marmiroli S. (2012). Environmental risk factors for amyotrophic lateral sclerosis: Methodological issues in epidemiologic studies. Ann. Ig..

