# Supplementary material for: Clinical and Lifestyle Factors and Risk of Amyotrophic Lateral Sclerosis: A Population-Based Case-Control Study
Source: Int J Environ Res Public Health. 2020 Jan 30;17(3):857. doi: 10.3390/ijerph17030857 (PMC7037077; doi:10.3390/ijerph17030857)
Supplement: Supplementary file 1 [file ijerph-17-00857-s001.pdf]

SUPPLEMENTAL MATERIAL

Table S1. Odds ratio (OR) with 95% confidence interval (CI) of ALS risk according to personal characteristics and clinical factors without subjects with a family history of ALS.

Factor	Cases (y/n)	Controls (y/n)	OR[a]	OR[b]	(95% CI)
**Personal information and habits**					
Dominant hand					
Right-handed	79	122	Ref.		
Left-handed	4	4	1.54	1.47	(0.35-6.24)
Ambidextrous	7	7	1.54	1.58	(0.52-4.76)
Non-right handed	11/79	11/122	1.54	1.54	(0.63-3.79)
Dominant foot					
Right-handed	79	121	Ref.		
Left-handed	10	10	1.53	1.57	(0.61-4.00)
Ambidextrous	1	2	0.77	0.78	(0.07-8.91)
Non-right handed	11/79	12/121	1.40	1.43	(0.59-3.45)
Regular use of skin cream[c]	25/41	64/68	0.65	0.56	(0.28-1.11)
Having or ever had amalgam fillings[c]	40/26	78/54	1.07	1.11	(0.59-2.10)
Regular use of chewing gum[c]	15/51	17/115	1.99	2.09	(0.92-4.74)
Eat fish[c]	47/19	118/14	0.29	0.30	(0.13-0.66)
Eat ≥3 fish-based meals per week[c]	6/60	18/114	0.63	0.60	(0.22-1.62)
Wine drinking[c]	42/48	84/49	0.51	0.50	(0.28-0.90)
Alcohol unit drinking[c,d]	10/56	21/111	0.94	1.01	(0.43-2.37)
Main source of drinking water					
No preference	34	45	Ref.	Ref.	
Municipal water	22	55	0.53	0.48	(0.24-0.95)
Private wells	4	3	1.76	1.38	(0.28-6.78)
Bottled water	30	30	1.32	1.15	(0.57-2.31)
Current use of any private well water	12/78	9/124	2.12	1.99	(0.79-4.99)
Have ever used a private well/fountain for drinking water	36/54	45/88	1.30	1.28	(0.72-2.29)
Current use of a private well/fountain for irrigation	14/76	30/103	0.63	0.59	(0.29-1.21)
Ever smoking	46/44	64/69	1.13	1.22	(0.68-2.17)
Current smoking	10/80	14/119	1.06	1.08	(0.44-2.66)
Use of dietary supplements in the former 20 years:	22/68	46/87	0.61	0.59	(0.32-1.08)
Vitamin supplements	13/77	29/104	0.61	0.60	(0.28-1.26)
Vitamin and mineral supplements	14/76	27/106	0.72	0.64	(0.31-1.32)
Aminoacidic supplements	3/87	2/131	2.26	2.37	(0.36-15.73)
Energy drinks	7/83	11/122	0.94	0.99	(0.34-2.83)
Selenium-containing supplements	21/69	36/97	0.82	0.85	(0.44-1.61)
**Clinical history**					
Any trauma requesting medical evaluation	33/57	49/84	0.99	1.13	(0.63-2.01)
Head trauma	17/73	13/120	2.15	2.44	(1.09-5.45)
Trunk trauma	9/81	8/125	1.74	1.81	(0.65-4.99)
Arm trauma	15/75	39/94	0.48	0.51	(0.26-1.02)
Any fracture	34/56	54/79	0.89	0.90	(0.52-1.58)
Head fracture	3/87	1/132	4.55	4.26	(0.42-43.27)
Trunk fracture	6/84	10/123	0.88	0.96	(0.33-2.84)
Arm fracture	26/64	45/88	0.79	0.79	(0.43-1.43)
Electric shock trauma	7/83	5/128	2.16	2.32	(0.68-7.86)
Previous Polio vaccine	39/51	65/68	0.80	0.78	(0.43-1.42)
Previous spinal anesthesia	23/67	35/98	0.96	0.98	(0.52-1.83)
Ever been blood donor	23/67	35/98	0.96	1.11	(0.58-2.12)

Any surgery	73/17	112/21	0.81	0.77	(0.37-1.60)
with general anesthesia	22/51	34/78	0.99	1.00	(0.52-1.92)

[a]Crude model; [b]Model adjusted by sex, age, and educational attainment; [c]Section missing for 25 subjects due to pilot version of the questionnaire. Analysis performed in 198 participants only (66 cases/132 controls); [d]Recommended alcohol units (two alcohol units in men, one unit in women) used as cutpoints.

Table S2. Odds ratio (OR) with 95% confidence interval (CI) of ALS risk according to leisure activities and other lifestyle factors without subjects with a family history of ALS.

Factor	Cases (y/n)	Controls (y/n)	OR[a]	OR[b]	(95% CI)
Hunting	4/86	6/127	0.98	1.13	(0.29-4.40)
Fishing	20/70	37/96	0.74	0.69	(0.33-1.43)
Using lead	16/74	32/101	0.68	0.65	(0.30-1.40)
Using lead in fishermen only	16/4	32/5	0.62	0.86	(0.15-4.75)
Painting	6/84	13/120	0.66	0.74	(0.26-2.10)
Use of oil paints	2/88	8/125	0.36	0.41	(0.08-2.06)
Use of oil paints in painters only	2/4	8/5	0.31	0.43	(0.04-4.90)
Model-making	7/83	8/125	1.32	1.51	(0.50-4.54)
Gardening	41/49	63/70	0.93	1.04	(0.60-1.80)
Any use of pesticides?	20/70	26/107	1.18	1.25	(0.64-2.44)
Using pesticides for gardening?	16/74	19/114	1.30	1.41	(0.67-2.99)
Using herbicides for gardening?	16/74	13/120	2.00	2.07	(0.92-4.62)
Using fungicides for gardening?	7/83	11/122	0.94	1.08	(0.40-2.97)
Photograph darkroom printing?	4/86	24/109	0.21	0.21	(0.07-0.65)
Play Sports	37/53	63/70	0.78	0.85	(0.45-1.60)
Play competitive sports	9/81	22/111	0.56	0.49	(0.21-1.17)
Play soccer	13/77	21/112	0.90	0.78	(0.34-1.81)
Play competitive soccer	5/85	6/127	1.25	1.05	(0.30-3.76)
Play volleyball	5/85	11/122	0.65	0.63	(0.20-2.00)
Play competitive volleyball	1/89	4/129	0.36	0.31	(0.03-2.92)
Cycling	4/86	7/126	0.84	0.88	(0.24-3.29)
Competitive cycling	1/89	4/129	0.36	0.34	(0.04-3.21)
Swimming	4/86	7/126	0.84	1.03	(0.26-4.01)
Competitive swimming	1/89	1/132	1.48	1.84	(0.11-31.29)
Skiing	4/86	7/126	0.84	1.31	(0.35-4.94)
Competitive skiing	0/90	0/135	-	-	
Play athletics	7/83	16/117	0.62	0.71	(0.27-1.85)
Competitive athletics	0/90	6/127	-	-	
Play tennis	3/87	7/126	0.62	0.81	(0.19-3.34)
Play competitive tennis	1/89	0/133	-	-	

[a]Crude model; [b]Adjusted by sex, age, and educational attainment; CI: confidence interval; OR: odds ratio.

Table S3. Odds ratio (OR) with 95% confidence interval (CI) of ALS risk according to personal characteristics and clinical factors in men.

Factor	Cases (y/n)	Controls (y/n)	OR[a]	OR[b]	(95% CI)
**Personal information and habits**					
Dominant hand					
Right-handed	44	65	Ref.		
Left-handed	3	2	2.22	2.33	(0.35-15.63)
Ambidextrous	4	4	1.48	1.46	(0.34-6.40)
Non-right handed	7/44	6/65	1.72	1.74	(0.53-5.72)
Dominant foot					
Right-handed	43	64	Ref.		
Left-handed	8	6	1.98	1.88	(0.59-6.00)
Ambidextrous	0	1	-	-	
Non-right handed	8/43	7/64	1.70	1.62	(0.53-4.94)
Regular use of skin cream[c]	7/32	20/51	0.56	0.47	(0.17-1.29)
Having or ever had amalgam fillings[c]	26/13	40/31	1.55	1.80	(0.75-4.32)
Regular use of chewing gum[c]	10/29	11/60	1.88	2.18	(0.72-6.58)
Eat fish[c]	30/9	66/5	0.25	0.28	(0.09-0.94)
Eat ≥3 fish-based meals per week[c]	3/36	12/59	0.41	0.37	(0.09-1.43)
Wine drinking[c]	29/22	56/15	0.35	0.36	(0.16-0.82)
Alcohol unit intake[c,d]	5/34	11/60	0.80	0.79	(0.24-2.62)
Main source of drinking water					
No preference	19	23	Ref.	Ref.	
Municipal water	12	29	0.50	0.48	(0.19-1.23)
Private wells	3	0	-	-	
Bottled water	17	19	1.08	0.95	(0.19-1.23)
Current use of any private well water	8/43	3/68	4.22	3.93	(0.96-16.13)
Have ever used a private well/fountain for drinking water	23/28	18/53	2.42	2.69	(1.18-6.12)
Current use of a private well/fountain for irrigation	9/42	12/59	1.05	0.94	(0.35-2.55)
Ever smoking	30/21	47/24	0.73	0.75	(0.35-1.62)
Current smoking	7/44	8&63	1.25	1.25	(0.40-3.90)
Use of dietary supplements in the former 20 years:	17/34	20/51	1.28	1.26	(0.55-2.87)
Vitamin supplements	10/41	9/62	1.68	1.89	(0.66-5.36)
Vitamin and mineral supplements	8/43	12/59	0.91	0.75	(0.26-2.11)
Aminoacidic supplements	3/48	1/70	4.37	2.98	(0.27-32.68)
Energy drinks	6/45	10/61	0.81	0.85	(0.26-2.73)
Selenium-containing supplements	13/48	14/57	1.39	1.44	(0.59-2.55)
**Clinical history**					
Any trauma requesting medical evaluation	19/32	30/41	0.81	0.86	(0.39-1.90)
Head trauma	12/39	7/64	2.81	3.35	(1.15-9.75)
Trunk trauma	4/47	3/68	1.93	2.21	(0.45-10.81)
Arm trauma	6/45	25/46	0.25	0.21	(0.08-6.61)
Any fracture	15/36	30/41	0.57	0.62	(0.28-1.37)
Head fracture	2/49	1/70	2.86	3.15	(0.26-38.38)
Trunk fracture	4/47	6/65	0.92	1.19	(0.29-4.82)
Arm fracture	9/42	25/46	0.39	0.39	(0.16-0.97)
Electric shock trauma	7/44	4/67	2.66	2.69	(0.72-10.07)
Previous Polio vaccine	26/25	38/33	0.90	0.81	(0.37-1.79)
Previous spinal anesthesia	12/39	25/46	0.57	0.61	(0.26-1.41)
Ever been blood donor	17/34	23/48	1.04	1.12	(0.50-2.54)
Any surgery	40/11	61/10	0.60	0.65	(0.23-1.79)
with general anesthesia	11/29	24/37	0.58	0.58	(0.24-1.43)

ALS cases in the family	4/47	0/71	-	-	

[a]Crude model; [b]Model adjusted by age, and educational attainment; [c]Section missing for 12 subjects due to pilot version of the questionnaire. Analysis performed in 122 participants only (39 cases/71 controls); [d]Recommended alcohol units (two alcohol units in men, one unit in women) used as cutpoints.

Table S4. Odds ratio (OR) with 95% confidence interval (CI) of ALS risk according to personal characteristics and clinical factors in women.

Factor	Cases (y/n)	Controls (y/n)	OR[a]	OR[b]	(95% CI)
**Personal information and habits**					
Dominant hand					
Right-handed	39	59	Ref.		
Left-handed	1	2	0.76	0.75	(0.06-9.24)
Ambidextrous	4	3	2.02	1.94	(0.39-9.50)
Non-right handed	5/39	5/59	1.51	1.46	(0.39-5.50)
Dominant foot					
Right-handed	40	59	Ref.		
Left-handed	3	4	1.11	1.19	(0.24-5.89)
Ambidextrous	1	1	1.48	1.37	(0.08-23.38)
Non-right handed	4/40	5/59	1.18	1.23	(0.31-4.97)
Regular use of skin cream[c]	18/11	46/17	0.60	0.59	(0.23-1.51)
Having or ever had amalgam fillings[c]	16/13	39/24	0.76	0.72	(0.28-1.84)
Regular use of chewing gum[c]	6/23	7/56	2.09	2.12	(0.62-7.21)
Eat fish[c]	18/11	54/9	0.27	0.25	(0.09-0.74)
Eat ≥3 fish-based meals per week[c]	3/26	6/57	1.10	1.12	(0.24-5.17)
Wine drinking[c]	14/30	29/35	0.56	0.55	(0.24-1.25)
Alcohol unit intake[c,d]	5/24	10/53	1.10	1.18	(0.35-4.02)
Main source of drinking water					
No preference	23	15	Ref.	Ref.	
Municipal water	26	12	0.71	0.67	(0.25-1.78)
Private wells	3	1	0.51	0.45	(0.04-5.08)
Bottled water	12	16	2.04	1.84	(0.63-5.32)
Current use of any private well water	4/40	6/58	0.97	0.90	(0.23-3.49)
Have ever used a private well/fountain for drinking water	15/29	28/36	0.67	0.56	(0.24-1.33)
Current use of a private well/fountain for irrigation	5/39	18/46	0.33	0.28	(0.09-0.85)
Ever smoking	19/25	18/46	1.94	2.14	(0.92-4.94)
Current smoking	4/40	7/57	0.81	0.79	(0.20-3.02)
Use of dietary supplements in the former 20 years:	9/35	26/38	0.38	0.35	(0.14-0.87)
Vitamin supplements	7/37	20/44	0.42	0.40	(0.15-1.07)
Vitamin and mineral supplements	8/36	15/49	0.73	0.68	(0.25-1.84)
Aminoacidic supplements	1/43	1/63	1.47	1.80	(0.10-33.74)
Energy drinks	1/43	1/63	1.47	1.33	(0.07-25.40)
Selenium-containing supplements	10/34	23/41	0.52	0.53	(0.21-1.33)
**Clinical history**					
Any trauma requesting medical evaluation	16/28	19/45	1.35	1.52	(0.65-3.55)
Head trauma	7/37	6/58	1.83	1.89	(0.58-6.16)
Trunk trauma	5/39	5/59	1.51	1.63	(0.43-5.17)
Arm trauma	19/34	14/50	1.05	1.11	(0.44-2.83)
Any fracture	19/25	24/40	1.27	1.26	(0.55-2.86)
Head fracture	1/43	0/64	-	-	
Trunk fracture	2/42	4/60	0.71	0.70	(0.12-4.12)
Arm fracture	17/27	20/44	1.39	1.39	(0.60-3.21)

Electric shock trauma	0/44	1/63	-	-	
Previous Polio vaccine	15/29	28/36	0.67	0.64	(0.26-1.56)
Previous spinal anesthesia	12/32	11/53	1.81	1.73	(0.66-4.54)
Ever been blood donor	7/37	14/50	0.68	0.72	(0.26-2.01)
Any surgery	36/8	52/12	1.04	0.93	(0.33-2.62)
with general anesthesia	12/24	11/41	1.82	1.80	(0.67-4.86)
ALS cases in the family	1/43	2/62	0.72	0.59	(0.05-7.22)

[a]Crude model; [b]Model adjusted by age, and educational attainment; [c]Section missing for 16 subjects due to pilot version of the questionnaire. Analysis performed in 92 participants only (29 cases/63 controls); [d]Recommended alcohol units (two alcohol units in men, one unit in women) used as cutpoints.

Table S5. Odds ratio (OR) with 95% confidence interval (CI) of ALS risk according to leisure activities and other lifestyle factors in men.

Factor	Cases (y/n)	Controls (y/n)	OR[a]	OR[b]	(95% CI)
Hunting	4/47	6/65	0.92	1.15	(0.29-4.63)
Fishing	18/33	36/35	0.53	0.50	(0.23-1.10)
Using lead	16/35	31/40	0.59	0.59	(0.27-1.29)
Using lead in fishermen only	16/2	31/5	1.19	1.30	(0.20-8.55)
Painting	5/46	10/61	0.66	0.81	(0.25-2.67)
Use of oil paints	2/49	5/66	0.54	0.71	(0.12-4.07)
Use of oil paints in painters only	2/3	5/5	0.67	0.96	(0.07-12.67)
Model-making	5/46	8/63	0.86	1.01	(0.29-3.50)
Gardening	22/29	36/35	0.74	0.85	(0.40-1.81)
Any use of pesticides?	12/39	15/56	1.15	1.12	(0.46-2.73)
Using pesticides for gardening?	10/41	12/59	1.20	1.18	(0.45-3.11)
Using herbicides for gardening?	10/41	8/63	1.92	1.96	(0.69-5.58)
Using fungicides for gardening?	4/47	7/64	0.78	0.81	(0.11-3.04)
Photograph darkroom printing?	5/46	19/52	0.30	0.32	(0.10-0.98)
Play Sports	28/23	45/26	0.70	0.66	(0.29-1.46)
Play competitive sports	8/43	18/53	0.55	0.41	(0.15-1.08)
Play soccer	14/37	21/50	0.90	0.67	(0.29-1.59)
Play competitive soccer	6/45	6/65	1.44	1.10	(0.32-3.78)
Play volleyball	1/50	4/67	0.34	0.27	(0.03-2.58)
Play competitive volleyball	0/51	3/68	-	-	
Cycling	5/46	7/64	0.99	1.02	(0.29-3.59)
Competitive cycling	1/50	4/67	0.34	0.31	(0.03-3.01)
Swimming	2/49	5/66	0.54	0.47	(0.08-2.91)
Competitive swimming	0/51	1/70	-	-	
Skiing	4/47	5/66	1.12	1.65	(0.38-7.20)
Competitive skiing	0/51	0/71	-	-	
Play athletics	5/46	12/59	0.53	0.61	(0.19-1.93)
Competitive athletics	0/51	4/67	-	-	
Play tennis	2/49	6/65	0.44	0.48	(0.09-2.65)
Play competitive tennis	1/50	0/71	-	-	

[a]Crude model; [b]Model adjusted by age, and educational attainment.

Table S6. Odds ratio (OR) with 95% confidence interval (CI) of ALS risk according to leisure activities and other lifestyle factors in women.

Factor	Cases (y/n)	Controls (y/n)	OR[a]	OR[b]	(95% CI)
Hunting	0/44	0/64	-	-	
Fishing	2/42	1/63	3.00	3.42	(0.38-41.58)
Using lead	0/44	1/63	-	-	
Using lead in fishermen only	0/2	1/0	-	-	
Painting	1/43	3/61	0.47	0.47	(0.04-4.97)
Use of oil paints	0/44	3/61	-	-	
Use of oil paints in painters only	0/1	3/0	-	-	
Model-making	2/42	0/64	-	-	
Gardening	20/24	29/39	1.01	1.07	(0.49-2.36)
Any use of pesticides?	8/36	13/751	0.87	0.90	(0.33-2.46)
Using pesticides for gardening?	6/38	9/55	0.96	1.03	(0.33-3.18)
Using herbicides for gardening?	6/38	5/59	1.86	1.89	(0.53-6.73)
Using fungicides for gardening?	3/41	5/59	0.86	0.95	(0.21-4.40)
Photograph darkroom printing?	0/44	5/59	-	-	
Play Sports	10/34	18/46	0.75	0.86	(0.31-2.35)
Play competitive sports	2/42	4/60	0.71	0.74	(0.12-4.66)
Play soccer	0/44	0/64	-	-	
Play competitive soccer	0/44	0/64	-	-	
Play volleyball	4/40	7/57	0.81	0.88	(0.22-3.49)
Play competitive volleyball	1/43	1/63	1.47	1.72	(0.09-33.55)
Cycling	0/44	0/64	-	-	
Competitive cycling	0/44	0/64	-	-	
Swimming	3/41	2/62	2.27	3.11	(0.43-22.66)
Competitive swimming	1/43	0/64	-	-	
Skiing	1/43	2/62	0.72	0.99	(0.08-12.60)
Competitive skiing	0/44	0/64	-	-	
Play athletics	2/42	4/60	0.71	0.79	(0.13-4.74)
Competitive athletics	0/44	2/62	-	-	
Play tennis	2/42	1/63	3.00	4.00	(0.33-49.13)
Play competitive tennis	0/44	2/62	-	-	

[a]Crude model; [b]Model adjusted by age, and educational attainment.

Table S7. Odds ratio (OR) with 95% confidence interval (CI) of ALS risk according to personal characteristics and clinical factors in the Northern Italy provinces of Modena, Novara and Reggio Emilia.

Factors	Cases (y/n)	Controls (y/n)	OR[a]	OR[b]	(95% CI)
**Personal information and habits**					
Dominant hand					
Right-handed	65	105	Ref.		
Left-handed	4	2	3.23	2.89	(0.49-16.94)
Ambidextrous	7	7	1.62	1.42	(0.47-4.28)
Non-right handed	11/65	9/105	1.97	1.73	(0.67-4.48)
Dominant foot					
Right-handed	65	105	Ref.		
Left-handed	10	7	2.31	2.02	(0.71-5.74)
Ambidextrous	1	2	-	-	
Non-right footed	11/65	9/105	1.97	1.73	(0.67-4.51)
Regular use of skin cream[c]	18/37	56/57	0.50	0.37	(0.17-0.82)
Having or ever had amalgam fillings[c]	33/22	71/42	0.89	0.84	(0.41-1.73)
Regular use of chewing gum[c]	14/41	17/96	1.93	2.04	(0.87-4.83)
Eat fish[c]	37/18	101/12	0.24	0.24	(0.10-0.57)
Eat ≥3 fish-based meals per week[c]	2/53	13/100	0.29	0.29	(0.06-1.38)
Wine drinking[c]	38/38	79/35	0.44	0.45	(0.23-0.85)
Alcohol unit intake[c,d]	9/46	20/93	0.91	0.91	(0.37-2.25)
Main source of drinking water					
No preference	26	38	Ref.	Ref.	
Municipal water	21	48	0.64	0.59	(0.28-1.23)
Private wells	3	1	4.38	3.33	(0.31-35.69)
Bottled water	26	27	1.41	1.26	(0.59-2.70)
Current use of any private well water	10/66	6/108	2.73	2.70	(0.92-7.95)
Ever use a private well/fountain for drinking water	34/42	39/75	1.56	1.57	(0.84-2.96)
Current use of a private well/fountain for irrigation	11/65	28/86	0.52	0.47	(0.21-1.04)
Ever smoking	38/38	62/52	0.84	0.91	(0.49-1.68)
Current smoking	5/71	14/100	0.50	0.50	(0.17-1.48)
Use of the following dietary supplements in the former 20 years:	22/54	39/75	0.78	0.75	(0.40-1.44)
Vitamin supplements	13/63	23/91	0.82	0.83	(0.38-1.81)
Vitamin and mineral supplements	13/63	25/89	0.73	0.66	(0.31-1.44)
Aminoacidic supplements	4/72	2/112	3.11	3.72	(0.57-24.30)
Energy drinks	6/70	11/103	0.80	0.82	(0.27-2.51)
Selenium-containing supplements	17/59	32/82	0.74	0.75	(0.37-1.53)
**Clinical history**					
Any trauma requesting medical evaluation	31/45	46/68	1.02	1.13	(0.61-2.10)
Head trauma	16/60	12/102	2.27	2.38	(1.01-5.51)
Trunk trauma	9/67	7/107	2.05	2.08	(0.71-6.04)
Arm trauma	14/62	38/76	0.45	0.47	(0.23-0.95)
Any fracture	27/49	45/69	0.84	0.89	(0.48-1.66)
Head fracture	2/74	0/114	-	-	
Trunk fracture	5/71	10/104	0.73	0.80	(0.25-2.52)
Arm fracture	20/56	37/77	0.74	0.75	(0.39-1.46)
Electric shock trauma	6/70	5/109	1.87	2.20	(0.61-7.87)
Previous Polio vaccine	34/42	57/57	0.81	0.77	(0.39-1.54)
Previous spinal anesthesia	21/55	33/81	0.94	0.96	(0.49-1.85)
Ever been blood donor	10/56	31/83	0.96	1.08	(0.53-2.18)

Any surgery	64/12	100/14	0.75	0.70	(0.29-1.68)
with general anesthesia	20/44	32/68	0.97	0.97	(0.49-1.95)
ALS cases in the family	3/73	2/112	2.30	2.04	(0.32-12.99)

[a]Crude model; [b]Model adjusted by sex, age, and educational attainment; [c]Section missing for 22 subjects due to pilot version of the questionnaire. Analysis performed in 168 participants only (55 cases/113 controls); [d]Recommended alcohol units (two alcohol units in men, one unit in women) used as cutpoints.

Table S8. Odds ratio (OR) with 95% confidence interval (CI) of ALS risk according to personal characteristics and clinical factors in the Southern Italy province of Catania.

Factors	Cases (y/n)	Controls (y/n)	OR[a]	OR[b]	(95% CI)
**Personal information and habits**					
Dominant hand					
Right-handed	18	19	Ref.		
Left-handed	0	2	-	-	
Ambidextrous	1	0	-	-	
Non-right handed	1/18	2/19	0.53	0.39	(0.02-7.80)
Dominant foot					
Right-handed	18	18	Ref.		
Left-handed	1	3	0.33	0.31	(0.02-5.04)
Ambidextrous	0	0	-	-	
Non-right footed	1/18	3/18	0.33	0.31	(0.02-5.04)
Regular use of skin cream[c]	7/6	10/11	1.28	3.00	(0.40-22.85)
Having or ever had amalgam fillings[c]	9/4	8/13	3.66	5.83	(0.76-44.42)
Regular use of chewing gum[c]	2/11	1/21	3.64	3.19	(0.17-59.35)
Eat fish[c]	11/2	19/2	0.58	0.22	(0.01-4.80)
Eat ≥3 fish-based meals per week[c]	4/9	5/16	1.42	0.89	(0.10-8.29)
Wine drinking[c]	5/14	6/15	0.89	0.53	(0.11-2.67)
Alcohol unit intake[c,d]	1/12	1/20	1.67	0.75	(0.03-16.65)
Main source of drinking water					
No preference	8	8	Ref.	Ref.	
Municipal water	3	7	0.43	0.20	(0.02-1.67)
Private wells	1	2	0.50	1.26	(0.07-21.70)
Bottled water	7	4	1.75	1.45	(0.16-13.31)
Current use of any private well water	2/17	3/18	0.71	1.03	(0.11-9.41)
Ever use a private well/fountain for drinking water	4/15	7/14	0.53	0.62	(0.13-3.01)
Current use of a private well/fountain for irrigation	3/16	2/19	1.78	1.74	(0.18-17.21)
Ever smoking	11/8	3/18	8.25	20.32	(1.32-311.71)
Current smoking	6/13	1/20	9.23	9.59	(0.78-118.07)
Use of the following dietary supplements in the former 20 years:	4/15	7/14	0.53	0.69	(0.14-3.36)
Vitamin supplements	4/15	6/15	0.67	1.00	(0.19-5.27)
Vitamin and mineral supplements	3/16	2/19	1.78	1.24	(0.16-9.69)
Aminoacidic supplements	0/19	0/21	-	-	
Energy drinks	1/18	0/20	-	-	
Selenium-containing supplements	6/13	5/16	1.48	1.51	(0.32-7.13)
**Clinical history**					
Any trauma requesting medical evaluation	4/15	3/18	1.60	2.48	(0.31-19.58)
Head trauma	3/16	1/20	3.75	7.28	(0.43-122.82)
Trunk trauma	0/19	1/20	-	-	
Arm trauma	2/17	1/20	2.35	5.64	(0.29-108.42)
Any fracture	7/12	9/12	0.78	0.80	(0.20-3.23)
Head fracture	1/18	1/20	1.11	0.60	(0.03-11.85)
Trunk fracture	1/18	0/21	-	-	
Arm fracture	6/13	8/13	0.75	0.91	(0.22-3.77)
Electric shock trauma	1/18	0/20	-	-	
Previous Polio vaccine	7/12	9/12	0.78	0.44	(0.10-1.96)
Previous spinal anesthesia	3/16	3/18	1.12	1.80	(0.26-12.56)
Ever been blood donor	4/15	6/15	0.67	1.13	(0.20-6.38)
Any surgery	12/7	13/8	1.05	0.88	(0.20-3.81)
with general anesthesia	3/9	3/10	1.11	1.81	(0.20-16.44)

ALS cases in the family	2/17	0/21	-	-

[a]Crude model; [b]Model adjusted by sex, age, and educational attainment; [c]Section missing for 6 subjects due to pilot version of the questionnaire. Analysis performed in 34 participants only (13 cases/21 controls); [d]Recommended alcohol units (two alcohol units in men, one unit in women) used as cutpoints.

Table S9. Odds ratio (OR) with 95% confidence interval (CI) of ALS risk according to leisure activities and other lifestyle factors in the Northern Italy provinces of Modena, Novara and Reggio Emilia.

**Factor**	**Cases (y/n)**	**Controls (y/n)**	**OR[a]**	**OR[b]**	**(95% CI)**
Hunting	2/74	6/108	0.49	0.49	(0.09-2.62)
Fishing	15/61	33/81	0.60	0.51	(0.23-1.14)
Using lead	11/65	28/86	0.52	0.46	(0.19-1.09)
Using lead in fishermen only	11/4	28/5	0.49	1.02	(0.15-6.90)
Painting	0/76	11/103	-	-	
Use of oil paints	0/76	7/107	-	-	
Use of oil paints in painters only	0/0	7/4	-	-	
Model-making	3/73	6/108	0.74	0.72	(0.16-3.15)
Gardening	34/42	61/53	0.70	0.78	(0.43-1.42)
Any use of pesticides?	17/59	27/87	0.93	0.98	(0.48-1.99)
Using pesticides for gardening?	13/63	20/94	0.97	1.06	(0.48-2.34)
Using herbicides for gardening?	13/63	12/102	1.75	1.86	(0.78-4.43)
Using fungicides for gardening?	6/70	11/103	0.80	0.95	(0.33-2.78)
Photograph darkroom printing?	5/71	24/90	0.26	0.26	(0.09-0.75)
Play Sports	29/47	54/60	0.69	0.71	(0.35-1.42)
Play competitive sports	9/67	18/96	0.72	0.66	(0.26-1.66)
Play soccer	11/65	19/95	0.85	0.73	(0.29-1.81)
Play competitive soccer	4/110	6/70	2.36	2.36	(0.60-9.27)
Play volleyball	4/72	8/106	0.74	0.67	(0.18-2.46)
Play competitive volleyball	1/75	4/110	0.37	0.29	(0.03-2.81)
Cycling	5/71	7/107	1.08	1.09	(0.31-3.84)
Competitive cycling	1/75	4/110	0.37	0.33	(0.03-3.11)
Swimming	4/72	6/108	1.00	1.16	(0.27-5.02)
Competitive swimming	0/76	1/113	-	-	
Skiing	5/71	7/107	1.08	1.79	(0.50-6.48)
Competitive skiing	0/76	0/114	-	-	
Play athletics	6/70	15/99	0.57	0.72	(0.25-2.03)
Competitive athletics	0/75	5/109	-	-	
Play tennis	3/73	7/107	0.63	0.74	(0.17-3.17)
Play competitive tennis	1/75	0/114	-	-	

[a]Crude model; [b]Model adjusted by sex, age, and educational attainment.

Table S10. Odds ratio (OR) with 95% confidence interval (CI) of ALS risk according to leisure activities and other lifestyle factors in the Southern Italy province of Catania.

Factor	Cases (y/n)	Controls (y/n)	OR[a]	OR[b]	(95% CI)
Hunting	2/17	0/21	-	-	
Fishing	5/14	4/17	1.52	0.57	(0.07-4.46)
Using lead	5/14	4/17	1.52	0.57	(0.07-4.46)
Using lead in fishermen only	5/0	4/0	-	-	
Painting	6/13	2/19	4.38	3.84	(0.41-36.24)
Use of oil paints	2/17	1/20	2.35	3.20	(0.15-68.27)
Use of oil paints in painters only	2/4	1/1	0.50	-[c]	
Model-making	4/15	2/19	2.53	1.26	(0.12-13.25)
Gardening	8/11	4/17	3.09	3.44	(0.70-16.84)
Any use of pesticides?	3/16	1/20	3.75	3.89	(0.28-54.59)
Using pesticides for gardening?	3/16	1/20	3.75	3.89	(0.28-54.59)
Using herbicides for gardening?	3/16	1/20	3.75	3.89	(0.28-54.59)
Using fungicides for gardening?	1/18	1/20	1.11	1.70	(0.07-39.99)
Photograph darkroom printing?	0/19	0/21	-	-	
Play Sports	9/10	9/12	1.20	1.09	(0.25-4.70)
Play competitive sports	1/18	4/17	0.24	0.21	(0.02-2.37)
Play soccer	3/16	2/19	1.78	0.66	(0.06-7.10)
Play competitive soccer	0/19	2/19	-	-	
Play volleyball	1/18	3/18	0.33	0.37	(0.03-4.98)
Play competitive volleyball	0/19	0/21	-	-	
Cycling	0/19	0/21	-	-	
Competitive cycling	0/19	0/21	-	-	
Swimming	1/18	1/20	1.11	2.05	(0.09-45.27)
Competitive swimming	1/18	0/21	-	-	
Skiing	0/19	0/21	-	-	
Competitive skiing	0/19	0/21	-	-	
Play athletics	1/18	1/20	1.11	0.74	(0.04-14.49)
Competitive athletics	0/19	1/20	-	-	
Play tennis	1/18	0/20	-	-	
Play competitive tennis	0/19	0/20	-	-	

[a]Crude model; [b]Model adjusted by sex, age, and educational attainment; [c]Too high estimate.

Table S11. Odds ratio (OR) with 95% confidence interval (CI) of ALS risk according to personal characteristics and clinical factors without carriers of *C9orf72* mutation.

Factor	Cases (y/n)	Controls (y/n)	OR[a]	OR[b]	(95% CI)
**Personal information and habits**					
Dominant hand					
Right-handed	77	124	Ref.		
Left-handed	4	4	1.61	1.53	(0.36-6.55)
Ambidextrous	7	8	1.84	1.85	(0.63-5.41)
Non-right handed	12/77	11/124	1.76	1.76	(0.74-4.18)
Dominant foot					
Right-handed	78	123	Ref.		
Left-handed	10	10	1.58	1.55	(0.60-4.00)
Ambidextrous	1	2	0.79	0.81	(0.07-9.20)
Non-right handed	11/78	12/123	1.45	1.42	(0.59-3.45)
Regular use of skin cream[c]	24/39	66/68	0.63	0.56	(0.28-1.12)
Having or ever had amalgam fillings[c]	38/25	79/55	1.06	1.13	(0.59-2.17)
Regular use of chewing gum[c]	14/49	18/116	1.84	1.98	(0.86-4.56)
Eat fish[c]	45/18	120/14	0.29	0.30	(0.13-0.67)
Eat ≥3 fish-based meals per week[c]	6/57	18/116	0.68	0.63	(0.23-1.73)
Wine drinking[c]	40/49	85/50	0.48	0.45	(0.25-0.82)
Alcohol unit drinking[c,d]	9/54	21/113	0.90	0.96	(0.40-2.33)
Main source of drinking water					
No preference	32	46	Ref.	Ref.	
Municipal water	21	55	0.55	0.48	(0.24-0.97)
Private wells	4	3	1.92	1.46	(0.30-7.19)
Bottled water	32	31	1.48	1.22	(0.61-2.46)
Current use of any private well water	10/79	9/126	1.77	1.67	(0.64-4.36)
Have ever used a private well/fountain for drinking water	36/53	46/89	1.31	1.29	(0.72-2.30)
Current use of a private well/fountain for irrigation	13/76	30/105	0.60	0.56	(0.27-1.18)
Ever smoking	46/43	65/70	1.15	1.23	(0.69-2.19)
Current smoking	10/79	15/120	1.01	1.03	(0.42-2.19)
Use of dietary supplements in the former 20 years:	24/65	46/89	0.71	0.68	(0.37-1.24)
Vitamin supplements	15/74	29/106	0.74	0.74	(0.36-1.51)
Vitamin and mineral supplements	15/74	27/108	0.81	0.72	(0.35-1.48)
Aminoacidic supplements	4/85	2/133	3.13	3.43	(0.56-21.16)
Energy drinks	7/82	11/124	0.96	1.02	(0.35-2.94)
Selenium-containing supplements	21/68	37/98	0.82	0.87	(0.45-1.66)
**Clinical history**					
Any trauma requesting medical evaluation	33/56	49/86	1.03	1.19	(0.66-2.14)
Head trauma	19/70	13/122	2.55	3.00	(1.35-6.67)
Trunk trauma	8/81	8/127	1.57	1.61	(0.57-4.59)
Arm trauma	14/75	39/96	0.46	0.48	(0.24-0.97)
Any fracture	33/56	54/81	0.88	0.90	(0.51-1.59)
Head fracture	3/86	1/134	4.67	4.37	(0.43-44.74)
Trunk fracture	6/86	10/125	0.90	1.02	(0.34-3.04)
Arm fracture	25/64	45/90	0.78	0.77	(0.42-1.41)
Electric shock trauma	6/83	5/130	1.88	1.90	(0.54-6.76)
Previous Polio vaccine	37/52	66/69	0.74	0.73	(0.40-1.33)
Previous spinal anesthesia	24/65	36/99	1.02	1.04	(0.56-1.94)
Ever been blood donor	23/66	37/98	0.92	1.06	(0.56-2.02)
Any surgery	73/16	113/22	0.89	0.86	(0.41-1.81)
with general anesthesia	23/50	35/78	1.03	1.04	(0.54-1.98)

[a]Crude model; [b]Model adjusted by sex, age, and educational attainment; [c]Section missing for 27 subjects due to pilot version of the questionnaire. Analysis performed in 197 participants only (63 cases/134 controls); [d]Recommended alcohol units (two alcohol units in men, one unit in women) used as cutpoints.

Table S12. Odds ratio (OR) with 95% confidence interval (CI) of ALS risk according to leisure activities and other lifestyle factors without carriers of *C9orf72* mutation.

Factor	Cases (y/n)	Controls (y/n)	OR[a]	OR[b]	(95% CI)
Hunting	4/85	6/129	1.01	1.15	(0.29-4.51)
Fishing	18/71	37/98	0.67	0.58	(0.28-1.23)
Using lead	14/75	32/103	0.60	0.53	(0.24-1.18)
Using lead in fishermen only	14/4	32/5	0.55	0.77	(0.14-4.22)
Painting	5/84	13/122	0.56	0.67	(0.22-2.02)
Use of oil paints	2/87	8/127	0.36	0.45	(0.09-2.24)
Use of oil paints in painters only	2/3	8/5	0.42	0.89	(0.05-14.74)
Model-making	7/82	8/127	1.36	1.58	(0.52-4.77)
Gardening	39/50	65/70	0.84	0.94	(0.54-1.63)
Any use of pesticides?	19/70	28/107	1.04	1.08	(0.55-2.12)
Using pesticides for gardening?	15/74	21/114	1.10	1.17	(0.56-2.47)
Using herbicides for gardening?	15/74	13/122	1.90	1.93	(0.85-4.38)
Using fungicides for gardening?	6/83	12/123	0.74	0.86	(0.30-2.47)
Photograph darkroom printing?	3/86	24/111	0.16	0.16	(0.04-0.57)
Play Sports	35/55	63/72	0.74	0.81	(0.42-1.53)
Play competitive sports	10/79	22/113	0.65	0.56	(0.24-1.30)
Play soccer	13/76	21/114	0.93	0.77	(0.33-1.81)
Play competitive soccer	6/83	6/129	1.55	1.27	(0.37-4.29)
Play volleyball	5/84	11/124	0.67	0.69	(0.22-2.18)
Play competitive volleyball	1/88	4/131	0.37	0.32	(0.03-3.00)
Cycling	5/84	7/128	1.09	1.11	(0.32-3.86)
Competitive cycling	1/88	4/131	0.37	0.33	(0.04-3.16)
Swimming	4/85	7/128	0.86	1.02	(0.24-4.13)
Competitive swimming	1/88	1/134	1.52	2.04	(0.12-34.38)
Skiing	5/84	7/128	1.09	1.78	(0.50-6.30)
Competitive skiing	0/89	0/135	-	-	
Play athletics	5/84	16/119	0.44	0.49	(0.17-1.44)
Competitive athletics	0/89	6/129	-	-	
Play tennis	3/86	7/128	0.64	0.78	(0.19-3.31)
Play competitive tennis	1/88	0/135	-	-	

[a]Crude model; [b]Adjusted by sex, age, and educational attainment; CI: confidence interval; OR: odds ratio.
